# RNA sequencing identifies clonal structure of T-cell repertoires in patients with adult T-cell leukemia/lymphoma

**DOI:** 10.1038/s41525-019-0084-9

**Published:** 2019-05-06

**Authors:** Amir Farmanbar, Robert Kneller, Sanaz Firouzi

**Affiliations:** 1 Department of Computational Biology and Medical Sciences, Graduate School of Frontier Sciences, The University of Tokyo, Tokyo, Japan; 2 Human Genome Center, The Institute of Medical Science, The University of Tokyo, Tokyo, Japan; 3 Research Center for Advanced Science and Technology, The University of Tokyo, Tokyo, Japan

**Keywords:** Genome informatics, High-throughput screening, Genome informatics, High-throughput screening

## Abstract

The diversity of T-cell receptor (TCR) repertoires, as generated by somatic DNA rearrangements, is central to immune system function. High-throughput sequencing technologies now allow examination of antigen receptor repertoires at single-nucleotide and, more recently, single-cell resolution. The TCR repertoire can be altered in the context of infections, malignancies or immunological disorders. Here we examined the diversity of TCR clonality and its association with pathogenesis and prognosis in adult T-cell leukemia/lymphoma (ATL), a malignancy caused by infection with human T-cell leukemia virus type-1 (HTLV-1). We analyzed 62 sets of high-throughput RNA sequencing data from 59 samples of HTLV-1−infected individuals—asymptomatic carriers (ACs), smoldering, chronic, acute and lymphoma ATL subtypes—and three uninfected controls to evaluate TCR distribution. Based on these TCR profiles, CD4-positive cells and ACs showed polyclonal patterns, whereas ATL patients showed oligo- or monoclonal patterns (with 446 average clonotypes across samples). Expression of TCRα and TCRβ genes in the dominant clone differed among the samples. ACs, CD4**-**positive samples and smoldering patients showed significantly higher TCR diversity compared with chronic, acute and lymphoma subtypes. CDR3 sequence length distribution, amino acid conservation and gene usage variability for ATL patients resembled those of peripheral blood cells from ACs and healthy donors. Thus, determining monoclonal architecture and clonal diversity by RNA sequencing might be useful for prognostic purposes and for personalizing ATL diagnosis and assessment of treatments.

## Introduction

The T-cell receptor (TCR), which is generated through random rearrangement of genomic V(D)J—variable(diversity)joining—segments, is the mediator of specific antigen recognition by T lymphocytes.^1,2^ This process is directly analogous to the generation of antibody diversity by somatic VDJ recombination of the B-cell receptor locus in B lymphocytes.^3^ The TCR consists of a heterodimer of two chains (αβ or γδ), both of which are products of V(D)J recombination.^4,5^ An individual’s TCR repertoire is shaped by biases during the process of VDJ recombination and by the subsequent expansion and deletion of certain T-cell clones upon antigen recognition during T-cell development in the thymus and later in the periphery.^3,6^ Because this somatic rearrangement occurs only in the T-cell genome and produces an extremely diverse repertoire of TCRs as a hallmark of the cellular adaptive immune system, it can be effectively used as a unique tag to enumerate and quantify T-cell clonality.^7,8^ The vast majority of TCR variation is within complementarity-determining region 3 (CDR3), which encompasses the VDJ recombination junctions and encodes the portion of the TCR that directly contacts peptide-bound major histocompatibility complex molecules.^1,9^ Thus, the sequence of CDR3 and the identity of the flanking V and J gene segments are widely used to classify TCR variants.^7^

The ability of human T cells to recognize a vast range of pathogens and initiate specific adaptive immune responses depends on the versatility of the TCR population.^10^ The clonotype of a TCR population is a molecular description of the unique sequences (typically described from the amino acid perspective) required to produce the antigen specificity of TCRs, as well as the specific V and J genes involved in the composite rearrangements.^11^ In normal individuals, the TCR repertoire is polyclonal and non-static.^12–14^ In contrast, clonal populations are a hallmark of malignancy, and clonal or oligoclonal populations of T and B cells can also occur in non-malignant conditions including human immunodeficiency virus and Epstein–Barr virus infections.^8^ Analysis of the TCR repertoire can thus characterize the host T-cell immune status in patients and normal individuals. In addition, quantitative assessment of T-cell clones can be of important prognostic significance to manage patients and their treatment.^15^

Using techniques such as Southern blot hybridization, polymerase chain reaction and flow cytometry, researchers have characterized T-cell proliferation.^16,17^ Recent next-generation sequencing (NGS) technologies have offered significant advantages over these earlier methods and have enabled extensive, comprehensive high-throughput measurement and analyses of the diversity of both TCRs and B-cell receptors (BCRs) to elucidate immune functions in health and disease.^7,18^ This approach has provided an estimation of approximately 10^6^ unique TCR sequences present in a human individual.^19^

There are several approaches for extracting CDR data from sequencing reads and determining the clonotype. One of the commonly used strategies for characterizing CDR3 sequences is TCR profiling, which amplifies cDNA or genomic DNA from the TCR β-chain CDR3 (β-CDR3) locus using predesigned PCR primers, followed by deep sequencing. Another approach, TCR profiling based on RNA sequencing (RNA-seq), is more informative, providing data from all transcribed genes present in the sample as well as enabling simultaneous analysis of TCRα, TCRβ, TCRγ and TCRδ, and has proven useful in personalized medicine.^15,20,21^

Adult T-cell leukemia/lymphoma (ATL) is an aggressive and complex malignancy that is caused by infection with human T-cell leukemia virus type-1 (HTLV-1) over a long latency period.^22–25^ An analysis of the clonality pattern and the number of clones based on the provirus integration sites indicates that the size of infected and leukemic clones is a determining factor for ATL development.^26–28^ High-throughput longitudinal analysis indicates that infected individuals with small clones and polyclonal patterns remain healthy over time, whereas those with large clones having an oligo- or monoclonal pattern develop ATL.^29,30^ Recently, genome-wide mutational spectra of large numbers of ATL cases have been published, and a list of frequently mutated genes in ATL has been proposed.^31^ Subsequently, clonal heterogeneity in ATL based on mutation profiles of cross-sectional, whole-exome sequencing samples has been monitored, and subclonal admixtures containing specific mutations have been proposed.^32^ In the current study, we focused on developing an understanding of clonal architecture based on TCR profiles in ATL. To our knowledge, this is the first report of TCR clonal architecture in ATL using RNA-seq.

## Results

**Fig. 1:**
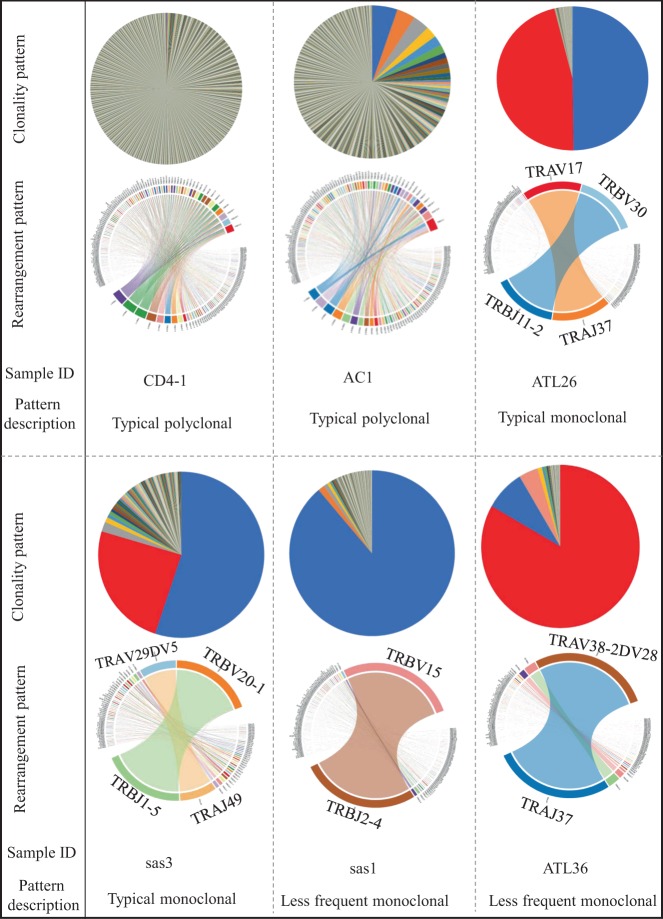
Representative examples of TCR α and β among samples. The pie charts (upper section) show the relative abundance of T-cell clones. The circus plots (lower section) show the rearrangements of the TCR chains. Complete sample information including ID abbreviations and TRA and TRB rearrangements is shown in Supplementary Table 1

Our study presents a detailed view of the TCR repertoire among 56 individuals with different subtypes of ATL, three ACs and three healthy donors (CD4-positive cells). Our results showed that the complexity and distribution of clonotypes differed among samples. ATL patients were more likely to have monoclonal TCRs, whereas healthy individuals and ACs had TCR subfamilies that had undergone polyclonal expansion. Monoclonally expanded TCRs showed different expression patterns of the TRA and TRB repertoire among different individuals (Fig. 1). Supplementary Fig. 1 shows the TCR profile of all analyzed samples. Based on the relative expression pattern of TRA and TRB, we divided the ATL samples into different groups. Group 1 included ATL patients (*N* = 30) with monoclonal T-cell expansion in which TRB expression was higher than that of TRA. Among the Group 1 samples, sas1 and ATL33 in particular had very little expression of TRA among the expanded T-cell population (Supplementary Fig. 1). Group 2 included ATL patients (*N* = 8) with monoclonal T-cell expansion in which TRA expression was greater than that of TRB. Among the Group 2 samples, sas9, ATL43 and ATL36 had very little expression of TRB among the expanded T-cell population. Group 3 included ATL patients (*N* = 10) with monoclonal T-cell expansion in which TRB and TRA had almost equal expression. Group 4 included ATL patients (*N* = 8) with a complex (i.e., non-monoclonal) pattern of T-cell expansion (Supplementary Fig. 1).

**Fig. 2:**
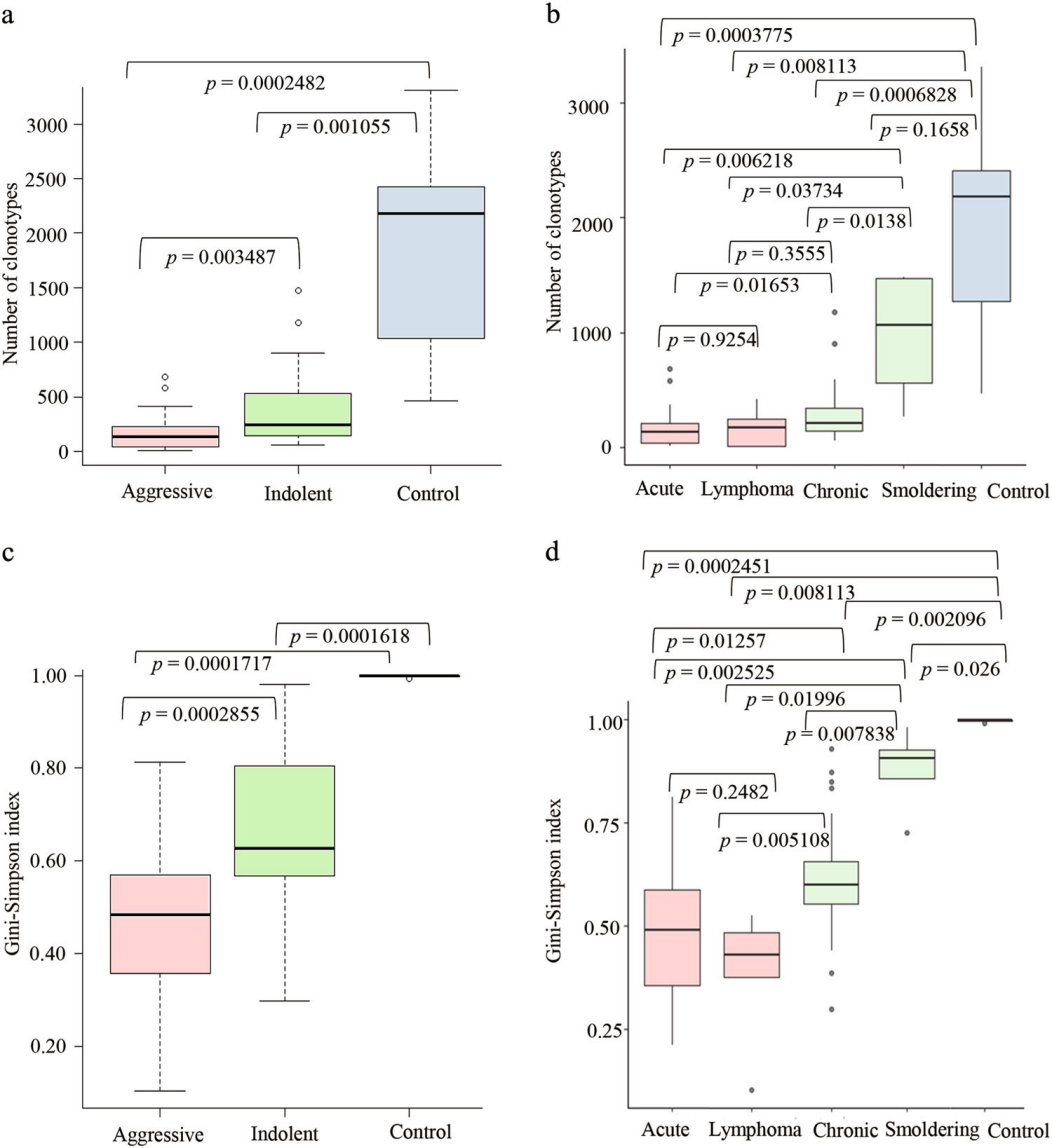
Analysis of the number and diversity of clonotypes present in individuals with different clinical subtypes of ATL. **a**, **b** The number of clonotypes was compared among the different groups of samples. **a** The number of clonotypes in aggressive ATL was significantly different from that in indolent ATL. The differences between the aggressive and indolent types as compared with the control group were also significant. **b** The number of clonotypes was compared among different ATL subtypes and the control samples. **c**, **d** Estimation of diversity was carried out with the Gini-Simpson index. **c** Gin–Simpson index values for aggressive ATL, indolent ATL and control samples were compared. **d** Gini–Simpson index values for different subtypes of ATL (acute, lymphoma, chronic and smoldering) and control samples were compared. **a–d** *p*-values were calculated by the Wilcoxon-Mann-Whitney (WMW*)* test using R. The control group consisted of ACs and normal CD4-positive samples. The differences between acute and lymphoma subtypes (for the number of clonotypes and the Gini-Simpson index) were not significant

In addition to the pattern of clonal expansion, we examined the number of detected clonotypes in each sample. The number of clonotypes ranged from 11 to 3309. The median and average number of clonotypes for ATL samples were 175 and 287, respectively, as compared with 1931 and 2179, respectively, for control samples (AC and CD4 cells) (Supplementary Table 1). The frequency distribution of clonotypes was notable for the number of clonotypes that was observed with low copy counts, suggesting that the TCR repertoire contains a large number of clones with a small clone size. There were significant differences in the number of clonotypes among different types and subtypes of ATL and control samples (Fig. 2a, b, Supplementary note 2: Supplementary Table-1, Supplementary Table-2) We estimated the clonotype diversity using the Gini-Simpson index. The Gini–Simpson index values for each sample are presented in Supplementary Table 1. There were significant differences in the estimated repertoire diversity between the control samples and ATL and among different subtypes of ATL (Fig. 2c, d) (Supplementary note 2: Supplementary Table-3, Supplementary Table-4). Details on the calculation of the *WMW* test are provided in Supplementary Note-2.

About 98% of the clonotypes across the samples expressed TCRs consisting of α and β chains, and ~2% of detected clonotypes consisted of δ and γ chains. The relative abundance of δ and γ chains in ATL samples was higher than that in AC and normal CD4 samples (Supplementary Fig. 2). Detailed information on TCR clonotypes is provided in Supplementary Table 1. The correlation between the number of clonotypes and the total sequencing reads from TCR was not linear (*R*^2^ = 0.0231), as shown in Supplementary Fig. 3.

**Fig. 3:**
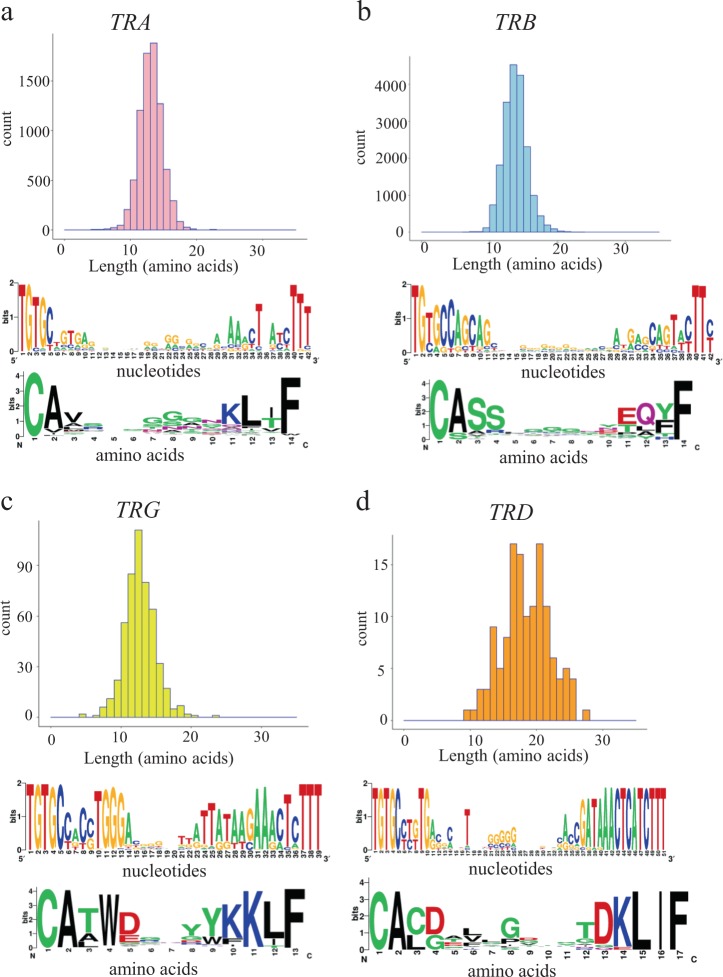
Length and amino acid conservation of the α, β, δ and γ chains of CDR3 sequences across samples. **a−d** Data are shown for CDR3 of (**a**) TRA, (**b**) TRB, (**c**) TRG and **d** TRD. The histogram (upper) shows the number of chains sequenced for a given length. We selected 14, 14, 13 and 17 amino acid lengths from each CDR3 of A, B, G and D, respectively, and performed WebLogo analysis. The *y* axis of each WebLogo plot shows the conservation score

We also quantified the number of shared nucleotide and amino acid sequences among repertoires, compared gene usage frequencies and estimated repertoire diversity among analyzed samples. β-CDR3 sequences had lengths ranging from 4 to 34 amino acids, with a median of 14 amino acids. α-CDR3 sequences had lengths ranging from 4 to 33 amino acids, with a median of 14 amino acids. δ-CDR3 sequences had lengths ranging from 10 to 28 amino acids, although they had a longer median of 19 amino acids. γ-CDR3 sequences had lengths ranging from 5 to 24 amino acids, although they had a shorter median of 13 amino acids (Fig. 3).

**Fig. 4:**
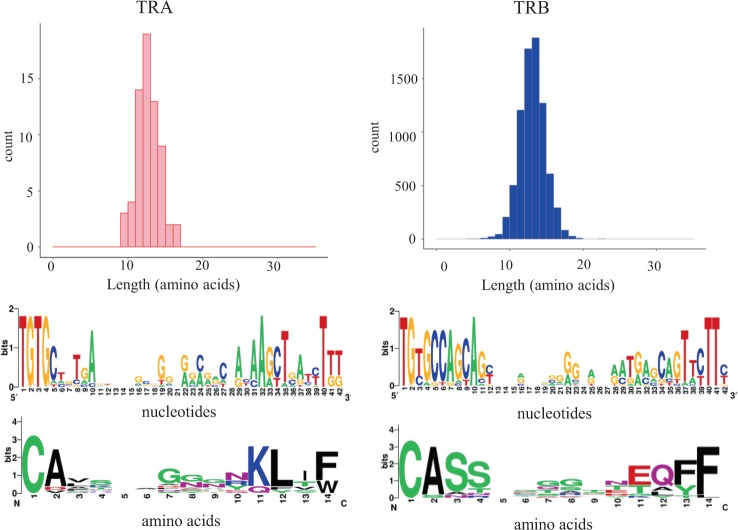
Length and amino acid conservation of α and β CDR3 sequences across samples among expanded clones. We analyzed expanded clones, i.e., those clonotypes with a frequency of >100. Data are shown as in Fig. 3

We also investigated whether the presence of any specific motif could provide prognostic information. The α-CDR3 and β-CDR3 sequences showed no strong over-representation of specific amino acids except for a slightly higher prevalence of glycine residues in the middle of the sequence logo. The prevalence of glycine residues was increased in more highly expanded clones (Fig. 4). In the case of the most highly expanded clones in ATL, the α-CDR3 glycines were further enriched at amino acid 7 and 8, and tryptophan (W) was enriched at aa 14 (Supplementary Fig. 4). Similarly, glycines in β-CDR3 were more enriched at aa 7 and 8, and phenylalanine (F) was enriched at aa 13 (Supplementary Fig. 5).

**Fig. 5:**
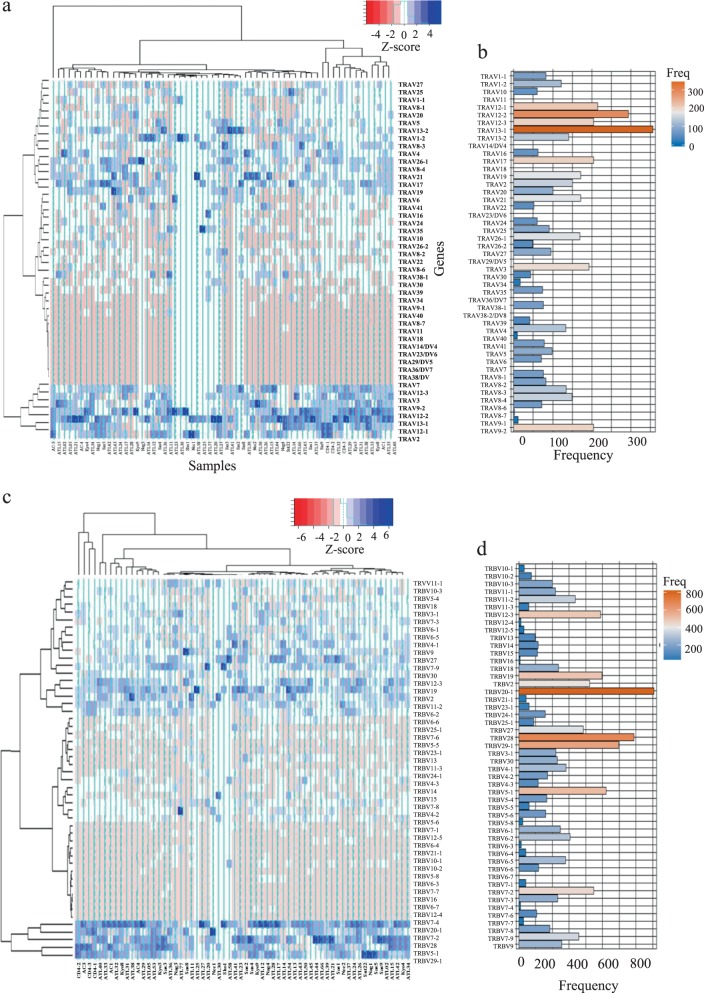
Analysis of TRA variable (TRAV) and TRB variable (TRBV) gene segment usage among analyzed samples. **a−d** Analysis of (**a**, **b**) TRAV and (**c**, **d**) TRBV gene segment usage, including (**a**, **c**) heatmap representation of TRAV and TRBV gene usage across samples and (**~**) frequency of observed TRAV and TRBV gene usages among the samples

There were skewed usages of TCR Vα and TCR Vβ subfamilies among the samples, particularly with respect to the expanded T-cell clones of patients. The five most abundant transcripts for each class corresponded to 13-1, 12-2, 12-1, 9-2 and 17 for TRAV genes and 20-1, 28, 29-1, 5-1 and 19 for TRBV genes (Fig. 5). TRAV 13-1 and TRBV 20-1 were the most abundant clonotypes among the samples (Fig. 5). We also compared TRAV and TRBV gene usage among CD4, ACs and ATL patients. TRAV 12-1, TRAV 19, TRBV 5-1, TRBV 29-1 and TRBV 7-2 were the most frequently observed clonotypes in the CD4-positive samples. TRAV 13-1, TRAV 12-1, TRAV 2, TRAV 12-2, TRBV 5-1 and TRBV 20-1 were the most frequently observed clonotypes in the AC samples. TRAV 13-1, TRAV 12-2, TRBV 20-1 and TRBV 28 were the most frequently observed clonotypes in the ATL samples. TRAV 13-1 and TRBV 20-1 were the most frequently observed clonotypes among the expanded clones in the ATL patients (Supplementary Figs. 6 and 7).

TRAJ and TRBJ gene usage was also examined. TRAJ 49, TRBJ 1-2 and TRBJ 2-1 were the most frequently observed clonotypes among ATL samples (Supplementary Figs. 8 and 9). TRAJ 4, TRAJ 23, TRBJ 2-7 and TRBJ 2-1 were the most frequently observed clonotypes in CD4-positive samples. TRAJ 37, TRAJ 49, TRAJ 39, TRAJ 4, TRAJ 42, TRBJ 12-1 and TRBJ 2-3 were the most frequently observed clonotypes in AC samples. TRAJ 49, TRAJ 37, TRBJ 2-1 and TRBJ 2-7 were the most frequently observed clonotypes in ATL samples. TRAJ 49, TRBJ 1-2 and TRBJ 2-1 were the most frequently observed clonotypes among the expanded clones in ATL patients (Supplementary Figs. 10 and 11). The TRDV-1 and TRGV-10 genes were used most frequently across the samples (Supplementary Fig. 12). We also investigated the presence of shared clonotypes at the nucleotide level across the samples (Supplementary Fig. 13).

TCRG expression was very low, <1% in 93% of patients except for four cases—Nag3, Sas4, Kyo8 and ATL15—which had a relative abundance for TCRG of 10.8%, 5.2%, 2.9%, and 1.97%, respectively. Nag3 was a 30-year-old patient with chronic ATL who had a proviral load of 52% (Supplementary Fig. 14).

Most of the analyzed samples showed a polyclonal pattern of BCRs (Supplementary Fig. 15). We examined the sequence length of CDR3 in IGH, IGK and IGL among individuals with ATL and ACs. CDR3 sequences of IGH, IGK and IGL had median lengths of 16, 11 and 13 amino acids, respectively. The motif analysis by WebLogo showed no significant difference between ATL and ACs (Supplementary Fig. 16). Gene usage analysis of the IGH segment showed that some V(D)J genes were used more frequently than others. IGHV3-23 was the most frequent clonotype among ACs. In contrast, among ATL patients, IGHV3-21 and IGHV3-30 were the most frequent clonotypes, in addition to IGHV3-23 (Supplementary Fig. 17).

ATL21 was a 76-year-old female with acute ATL and a 26.99% proviral load. The sample from this patient was exceptional in that it manifested a B-cell clonal expansion with the IGKV3-20/J4 clonotype. T-cell expansion in this individual was monoclonal with TRBV4-2 and TRAV13-1 (Supplementary Fig. 18).

We also found that in two cases (Sas9 and ATL77) TCRα and TCRβ of the dominant clone contained a non-synonymous point mutation. Sas9 had two top clones with identical rearrangement of TRAV12-2. At the nucleotide level, there was a mutation at the twenty-ninth base pair that substituted adenine (A) with guanine (G). As a result, at the amino acid level, the tenth amino acid was converted from asparagine (N) to serine (S) (Supplementary Fig. 19). ATL77 had two top clones with identical rearrangement of TRBV4-2. At the nucleotide level there was a mutation at the 28 base pair that substituted adenine (A) with guanine (G). As a result, at the amino acid level, the tenth amino acid was converted from arginine (R) to glycine (G) (Supplementary Fig. 20).

## Discussion

As we move toward an era of personalized medicine for ATL and other malignancies, there is an increasing demand for simple, quantitative and reproducible measures enabling the characterization and tracking of cancer cells and their associated microenvironment. Considering the inevitable role of immune system cells in the development, the control and/or even the treatment of malignancies, profiling of immune cell populations is of great importance. T-cell/B-cell clonality analysis has important clinical and research value, as it provides a specific and reproducible assessment of clonal diversity in lymphoproliferative disorders and can be used to characterize the repertoire of tumor-infiltrating T cells in solid tumors and to assess residual disease post-therapy in lymphoid malignancies.^7,21^ Many T-cell malignancies are characterized by extreme clonal skewing, with oligoclonal or even monoclonal T-cell expansions. Because TCR skewing arises in a predictable fashion following an antigen-specific response, monitoring TCR oligoclonality and tracking specific TCR clonotypes linked to malignancy, or other immunological disorders, have proven beneficial in the clinical setting.^33,34^ Characterization of the immune microenvironment using both the TCR and BCR repertoire and analyses to identify neo-antigenic immune targets provide a wealth of information in many cancer types and have prognostic value.^35,36^ In the current study, exploiting the capacity of high-throughput sequencing, we could fulfill our aim to analyze multiple samples from ATL individuals using RNA-seq data to enumerate, quantify, assess and understand the heterogeneity of TCR clones in ATL development.

Numerous studies have described how the TCR and BCR repertoires are altered in the context of certain infections, malignancies or immunological disorders.^33,37–39^ The emergence of specific TCR clonotypes or of an oligoclonal/monoclonal-skewed TCR repertoire is an important immunological ‘signature’.^34^ Recently, Thorsson et al.^35^ extensively analyzed TCR and BCR repertoires from RNA-seq data of various cancer types from The Cancer Genome Atlas cohort. They showed that higher TCR diversity correlates with an improved progression-free interval for a few tumor types (urothelial bladder carcinoma, colon adenocarcinoma, liver hepatocellular carcinoma and uterine corpus endometrial carcinoma).^35^ In the case of ATL, skewed TCR diversity was observed among almost all aggressive and indolent subtypes. We demonstrated that ACs and smoldering patients had higher TCR diversity; however, as the disease becomes more advanced and reaches a chronic, acute or lymphoma status, the TCR diversity significantly decreased (Fig. 2, Supplementary Table 1). This analysis has highlighted a strong correlation between tumor TCR diversity and ATL progression.

Among the ATL cases analyzed here, monoclonally expanded T-cell populations mainly manifested TCRαβ rearrangements. Whereas clonal expansion of both TCRVA and TCRVB was most frequently observed in our analyzed cases of ATL, expansion of one or the other was missing in some cases. Several cases showed dominant expression of only CDR3α or CDR3β. In other words, a dominant clone based on TCRα transcripts, but not TCRβ transcripts (or vice versa), was observed in some cases. For surface expression and functionality, each TCR heterodimer must be noncovalently bonded to a CD3 subunit. The TCR-CD3 complex serves a critical role in the differentiation, survival and function of T cells.^40^ Thus, this distorted expression pattern that leads to incomplete complex formation can be considered abnormal. This abnormal TCR gene expression in the malignant clone may explain the frequent loss or very low expression of surface CD3 in ATL.^41^ Consistent with our results, a similar phenomenon was reported by Gong et al. in the case of angioimmunoblastic T-cell lymphoma.^42^ Also, considering that mutations involved in activating TCR signaling have been observed in ATL,^31^ these mutations may allow for signaling for survival and proliferation in the absence of functional TCRs in such ATL cases. The mechanism responsible for abnormalities in TCR expression is of interest and needs to be further investigated.

Generally, on the basis of sequence count, γδ T cells account for 0.5−5% of the total T-cell population; however, the γδ T-cell fraction varies among cancer types.^21^ Consistent with previous observations, we found that only 2% of the T-cell population among these individuals consisted of γδ T cells. In some cases, such as Nag3, sas4 and kyo8, relatively expanded TCRG clones were evident. The reason for this expansion needs to be examined.

The presence of skewed clonal proliferation, and/or certain preferential V-segment usage in TCRs or BCRs has been reported in several B-cell and T-cell malignancies such as chronic lymphocytic leukemia,^43^ hairy cell leukemia,^44^ mantle cell lymphoma,^45^ and acute T lymphoblastic leukemia.^37^ In the case of ATL, although we demonstrated skewed TCR clonal proliferation across all samples, TCR gene usage was not found to be highly specific; TCRBV13-1 and TCRBV20-1 were frequently observed among the samples, the latter of which probably was not specific to ATL. We noted a similarly high prevalence of *TRBV20-1* usage in our control samples, as well as in normal human peripheral blood leukocytes as reported.^18^ Our results suggest that TRBV and TRAV gene usage in individuals with ATL does not deviate substantially from that of ACs or normal leukocytes.

There is relatively little similarity in TCR usage across different tumor types, highlighting the large degree of diversity in T cells in T-cell leukemia/lymphoma and/or tumor-infiltrating lymphocytes in other types of malignancies. In addition, among peripheral blood samples from normal individuals, shared clonotypes are very rare.^18,19^ Here we assessed the co-occurrence of CDR3 α and β chains to determine the frequency of patients with identical TCRs. We confirmed that, in ATL, enriched TCR sequences are typically unique to each patient and coincidental identification of cross-patient sequences is generally rare. The observed shared clonotypes belonged to low-abundance sequences that are unlikely to be tumor specific. Previous findings from several studies are consistent with our observation.

The main goal of this study was the analysis of TCR repertoires because it has been known that HTLV-1 infection leads to clonal expansion among infected T cells. Moreover, it is of note that HTLV-1 can infect different cell types including B cells.^46,47^ To our knowledge, the presence of clonal expansion among infected cells other than T cells has never been examined. Our approach has thus enabled the simultaneous investigation of clonal expansion in both T cells and B cells. We did not, however, generally observe B-cell clonal expansion in ATL. In almost all samples, the BCRs were polyclonal. Simultaneous expansion of TCRs and BCRs had occurred in only one patient, ATL21. This was a very rare case, but why this phenomenon happened will need to be clarified in future studies.

As RNA-seq has increased the resolution of the αβ T-cell receptor repertoire, TCR-based diagnoses have become more practical.^34^ Dissecting the distribution of TCR clonotypes within an individual, and across individuals, in ACs and different disease subtypes is critical to our understanding of ATL. Without using patient-specific or consensus degenerate primers and dedicated sequencing experiment, we were able to fully characterize the clonal architecture in ATL samples. Clonality data observed based on TCR variants were completely consistent with clonality analysis based on provirus integration sites.^29,30^ Considering that a single RNA-seq assay can simultaneously yield gene expression and TCR profiles, it may one day be performed as a general test to capture multidimensional, clinically relevant data for patients with ATL. Deciphering the heterogeneity of the TCR repertoire in tumors may have important implications for biomarker discovery in immunotherapy studies in different types of cancers, including ATL.

Our findings provide the first objective data for assessing and unraveling the complexity of T-cell clonality in ATL. The integrated high-throughput analysis used here to simultaneously evaluate TCRαβ repertoires has provided new insights into the structure and function of T-cell expansion in ATL. We have demonstrated that RNA-seq is a useful approach for determining clonality in ATL. This approach can be used for monitoring minimum residual disease after chemotherapy, immunotherapy and bone marrow transplantation, as well as longitudinal tracking of the disease status over time. Our findings may lead to biomarker discovery, the monitoring of changes in T-cell repertoires, prediction of clinical outcomes of patients and the evaluation of factors responsible for a variety of human immune responses in ATL.

## Methods

### ATL samples

We used data from 62 samples consisting of three healthy donors, three ACs and 56 individuals with different subtypes of ATL (smoldering, *N* = 5; chronic, *N* = 24; acute, *N* = 22; lymphoma, *N* = 5) deposited in the European Genome-phenome Archive (EGA) with accession number EGAD00001001410^31^. Diagnosis and subtype classification were based on the World Health Organization classification and the International Consensus Meeting Proposal.^48^ The initial human clinical samples were obtained from peripheral blood cells of Japanese individuals (ages: 30−86 years, average: 65.8 years). Human peripheral blood CD4-positive T cells (purchased from Lonza) were used as a control. RNA extraction, library preparation and subsequent sequencing were as described.^31^ In brief, RNA was extracted using RNeasy Mini kit (Qiagen), and RNA-seq Libraries were prepared using the NEBNext Ultra RNA Library Prep kit for Illumina (New England BioLabs) following the manufacturer’s instructions. The resulting libraries were subsequently sequenced by the Illumina HiSeq 2000 or 2500 platform with a standard 100-bp paired-end read protocol. The information regarding the samples is included in Supplementary Table S1.

### Bioinformatics pipeline

Unlike conventional common pipelines for the analysis of RNA-seq data that yield differential expression profiles, the alternative pipeline that was used in this study enables simultaneous analysis and quantification of TCR and B-cell receptor (BCR) profiles. Our pipeline takes advantage of three main tools MiXCR,^49^ VDJtools^50^ and tcR,^51^ which enable the profiling and measurement of clonotypes, statistical analysis and visualization of the results.

We used the raw whole transcriptome sequencing (total RNA-seq) data in FASTQ format as the input for the analysis. The input data underwent several pre-processing and post-processing steps to generate the final text (tab delimited) and visual outputs. In the pre-processing step, the alignment and assembly of the RNA-seq reads and the exporting of the clonotypes were conducted using MiXCR.^49^ MiXCR is a universal tool (it works on multiple platforms and with multiple forms of data) that enables straightforward simultaneous mining of TCR and BCR sequencing data from immune repertoire profiling in a single experiment. MiXCR has no dependency on any external software and does not require initial processing of the input file by the operator. MiXCR is not restricted with respect to the maximum size of the input file (i.e., the number of sequencing reads). MiXCR accepts different lengths for the sequencing input, but 100-bp paired-end libraries result in a higher number of clonotypes as compared with single-end libraries and those with shorter read lengths. Quality control procedures including filtering and error correction steps are also built into the analysis pipeline.^49,52^

We performed CDR3 extraction and gene segment alignment using MiXCR with default parameters based on the developers’ instructions (https://github.com/milaboratory/mixcr/). In brief, sequencing reads were aligned to reference V, D, J and C genes of T- or B-cell receptors. Then, the aligned reads were assembled to extract CDR3 gene regions. Finally, the clonotypes were exported in a human-readable/parsable tab-delimited text file format.^49^ Naming of the TCR/BCR gene**s** (gene features) was performed based on the international ImMunoGeneTics (IMGT) information system described in.^53^ In the post-processing steps, we performed further analysis, which consisted of computing various statistics and carrying out visualization with VDJtools^50^ and tcR.^51^ The comprehensive user documentation and source codes for VDJtools and tcR are accessible through https://github.com/mikessh/vdjtools and https://github.com/imminfo/tcr, respectively. Using these tools, we generated basic sample statistics—such as the read counts, number of clonotypes, frequency of the share of clonotypes in each sample, repertoire richness and diversity, segment usage heatmap and hierarchical clustering for samples. We also calculated the spectratype, which is a histogram of read counts based on CDR3 nucleotide/amino acid length, drew circos-style plots for V-J usage and determined the frequency of various V-J junctions. Sequence logos (a graphical representation of an amino acid or nucleic acid multiple sequence alignment) were generated using WebLogo^54^ (http://weblogo.berkeley.edu/logo.cgi). WebLogo is a motif visualization tool that generally takes a set of aligned sequences as input, calculates the weight (frequency or statistical significance) of each letter at each position and generates logo plots as output. We performed computational analyses using the Supercomputer System at the Human Genome Center, The Institute of Medical Science, The University of Tokyo. Further details on methodology and the analysis pipeline are provided in Supplementary Note-1: Supplementary methodology.

### Statistical analysis

Statistical analysis was performed using R Software (R version 3.5.0). Comparisons between two groups were conducted by a nonparametric statistical test, the Wilcoxon–Mann–Whitney (WMW) exact test, using R for calculations. Details on calculation of the WMW test are provided in Supplementary Note-2. For diversity estimations, the Gini-Simpson index was measured with the gini.simpson function of tcR^51^ package (a platform-independent R tool for statistical computing with respect to the advanced analysis of immune receptor repertoires). The coefficient of determination (*R*^2^) and Z-score were computed by R and the heatmap.2 function of the gplots R package in the VDJtools framework^50^ (which calculates a wide range of statistics for the analysis of immune repertoires), respectively.

### Ethics approval

Written informed consent was obtained from all participants, and access to the raw sequencing data was approved based on an agreement between Kyoto University and The University of Tokyo.

### Reporting summary

Further information on research design is available in the Nature Research Reporting Summary linked to this article.

## Supplementary information

Supplementary Information

Reporting Summary

## Data Availability

The datasets analyzed during the current study are available in the EGA repository (https://www.ebi.ac.uk/ega/datasets/EGAD00001001410).

## Supplementary information

**Supplementary information** accompanies the paper on the *npj Genomic Medicine* website (10.1038/s41525-019-0084-9).
