# Supplementary material for: RNA sequencing identifies clonal structure of T-cell repertoires in patients with adult T-cell leukemia/lymphoma
Source: NPJ Genom Med. 2019 May 6;4:10. doi: 10.1038/s41525-019-0084-9 (PMC6502857; doi:10.1038/s41525-019-0084-9)
Supplement: Supplementary file 1 — Supplementary Information [file 41525_2019_84_MOESM1_ESM.pdf]

Supplementary Information

RNA-sequencing identifies clonal structure of the T-cell repertoires in adult T-cell leukemia/lymphoma

- **Supplementary Figures (1~20)**
- **Supplementary Table 1**
- **Supplementary Note 1**
- **Supplementary Note 2**

Supplementary Figure1

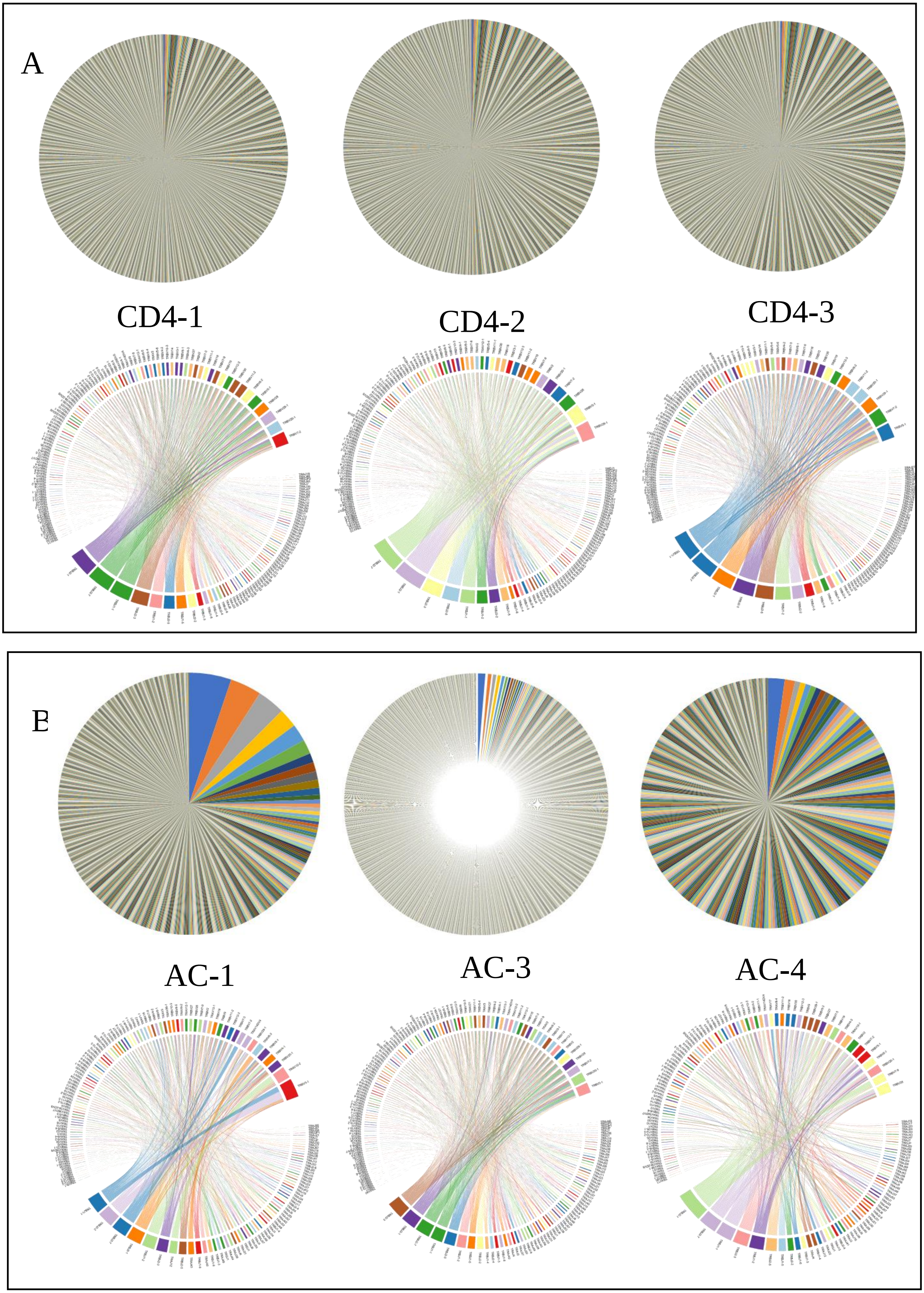

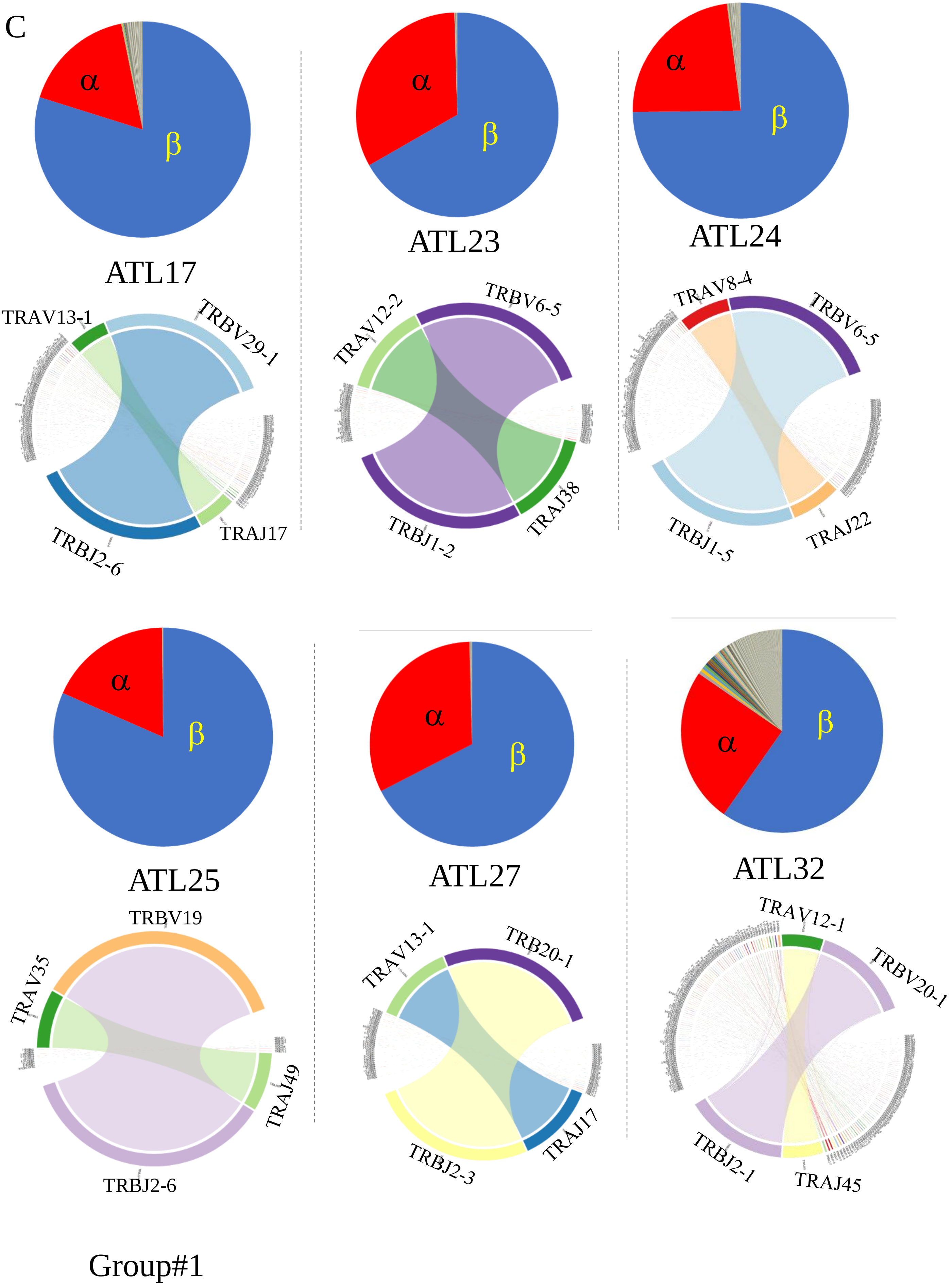

Supplementary Figure1-continue

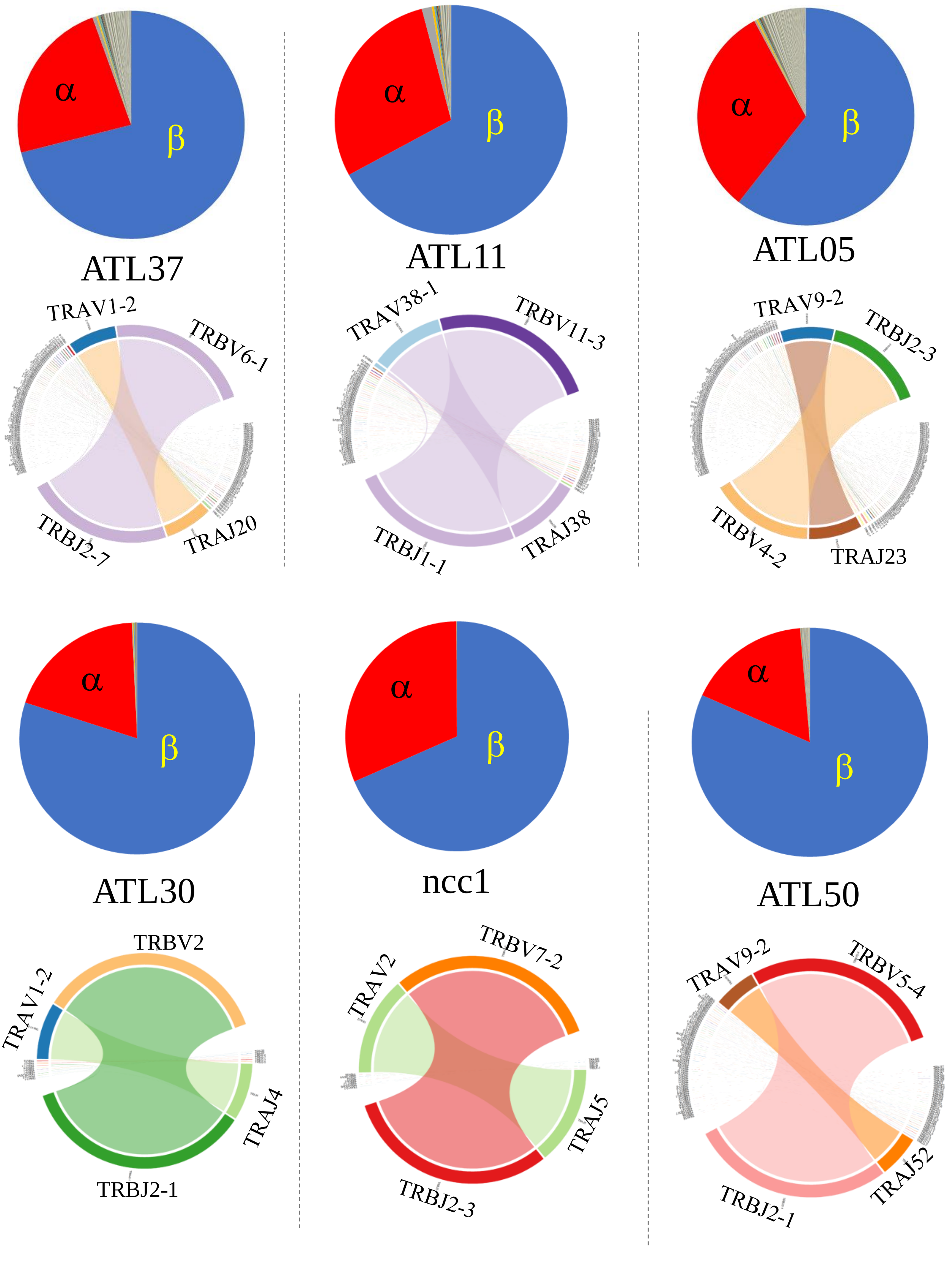

Group#1

Supplementary Figure1-continue

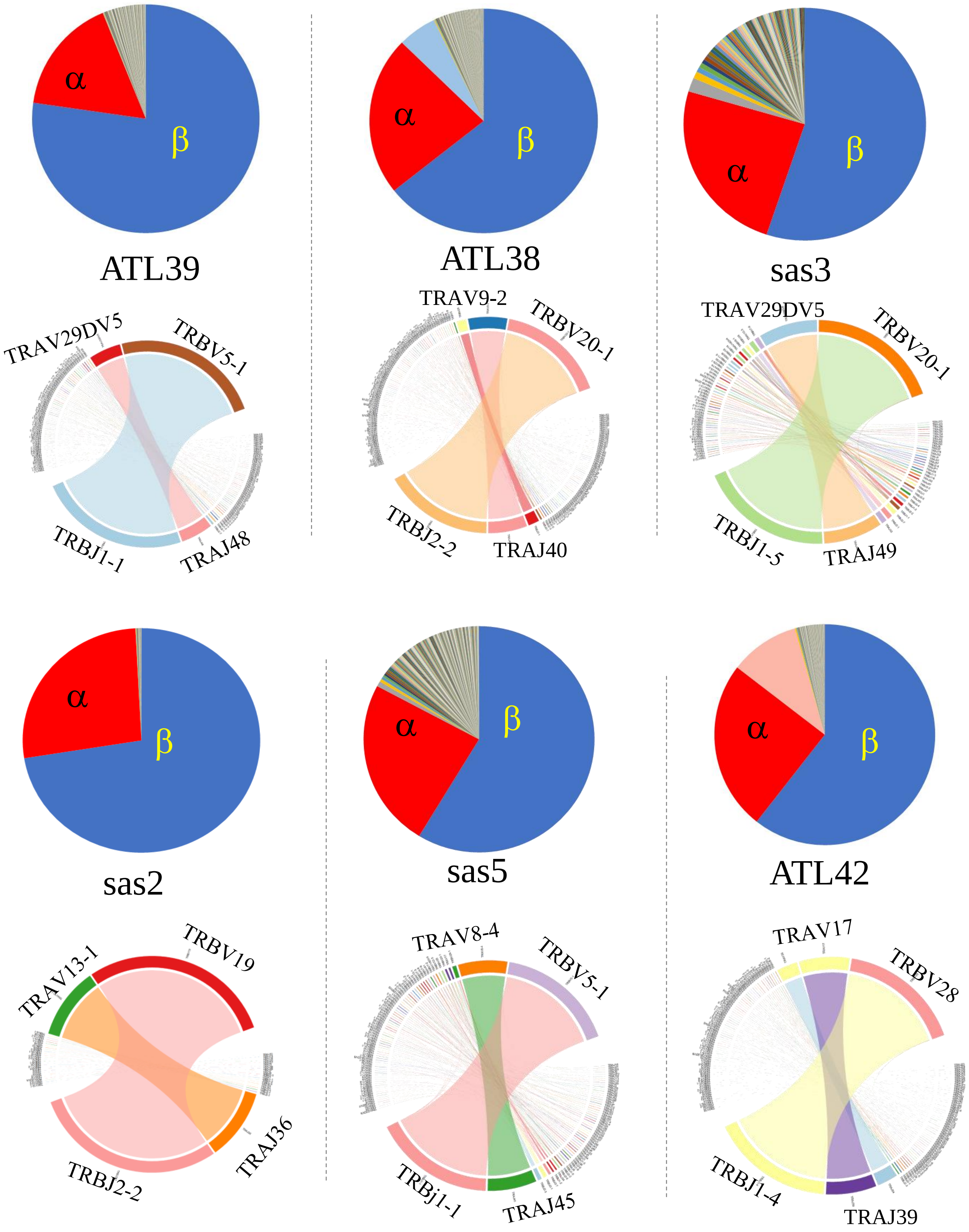
α
β
ATL39
TRAV29DV5
TRBV5-1
TRBJ1-1
TRAJ48
α
β
ATL38
TRAV9-2
TRBV20-1
TRBJ2-2
TRAJ40
α
β
sas3
TRAV29DV5
TRBV20-1
TRBJ1-5
TRAJ49
α
β
sas2
TRAV13-1
TRBV19
TRBJ2-2
TRAJ36
α
β
sas5
TRAV8-4
TRBV5-1
TRBj1-1
TRAJ45
α
β
ATL42
TRAV17
TRBV28
TRBJ1-4
TRAJ39

Group#1

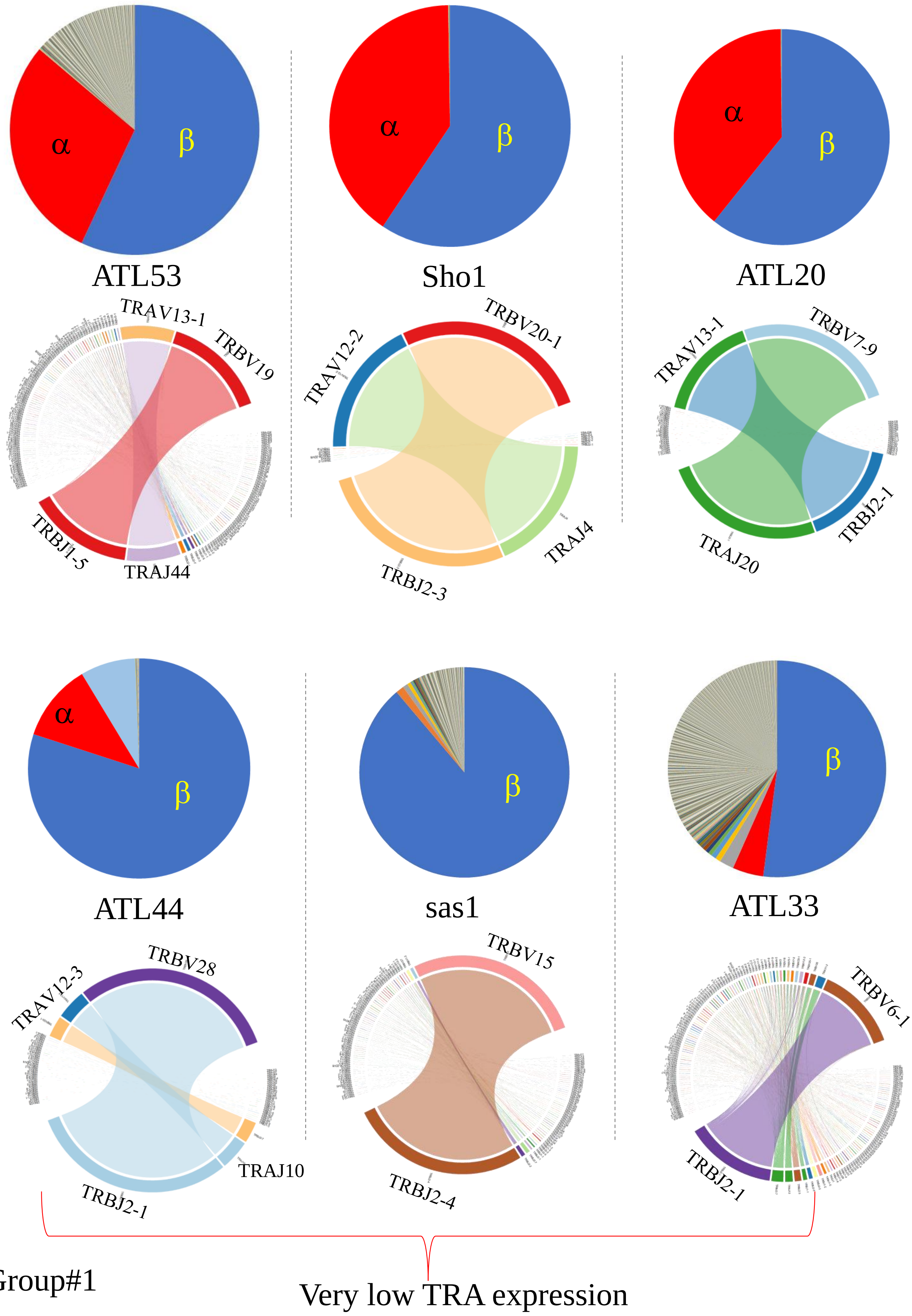

Supplementary Figure1-continue

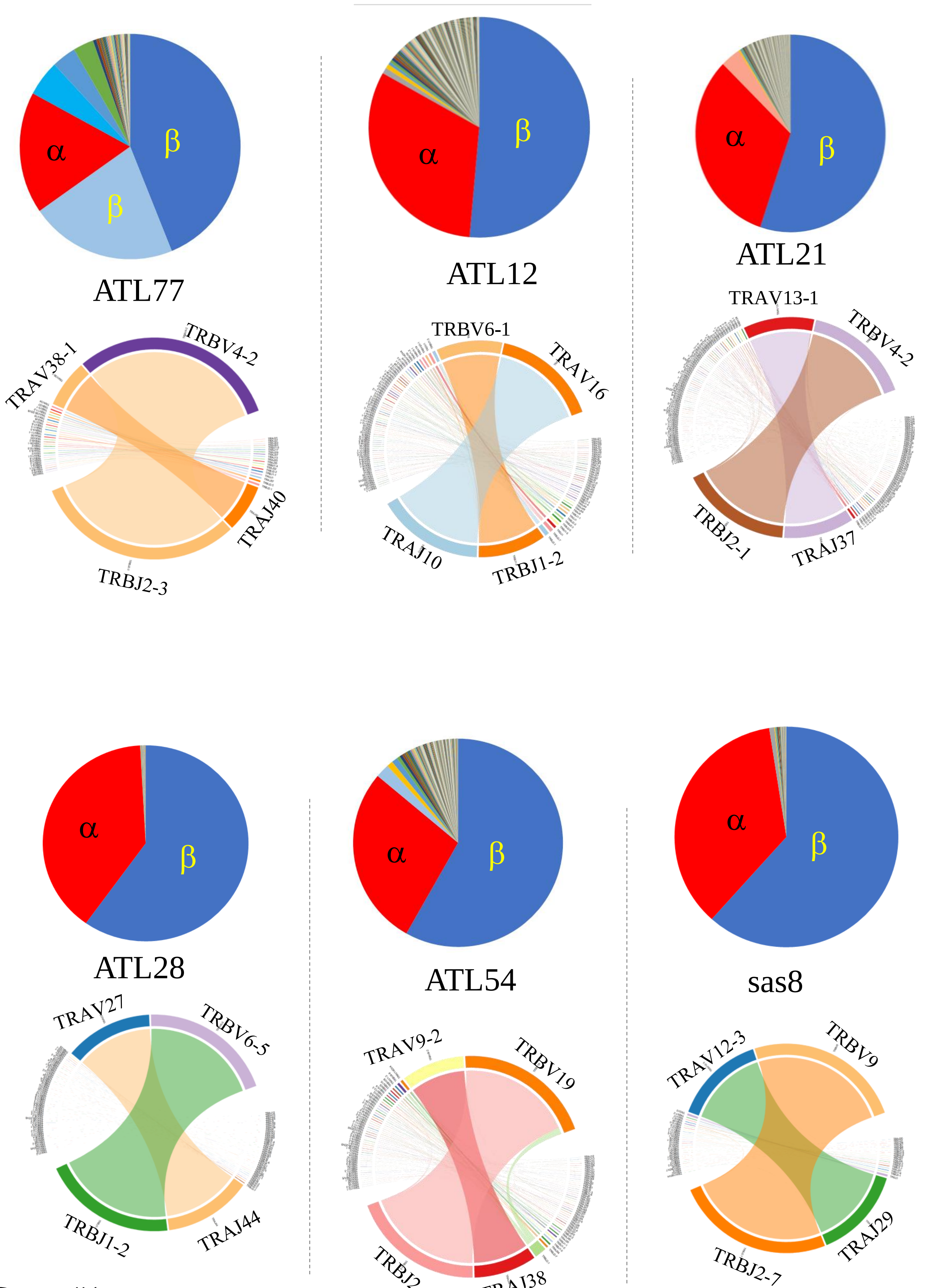

Group#1

Supplementary Figure1-continue

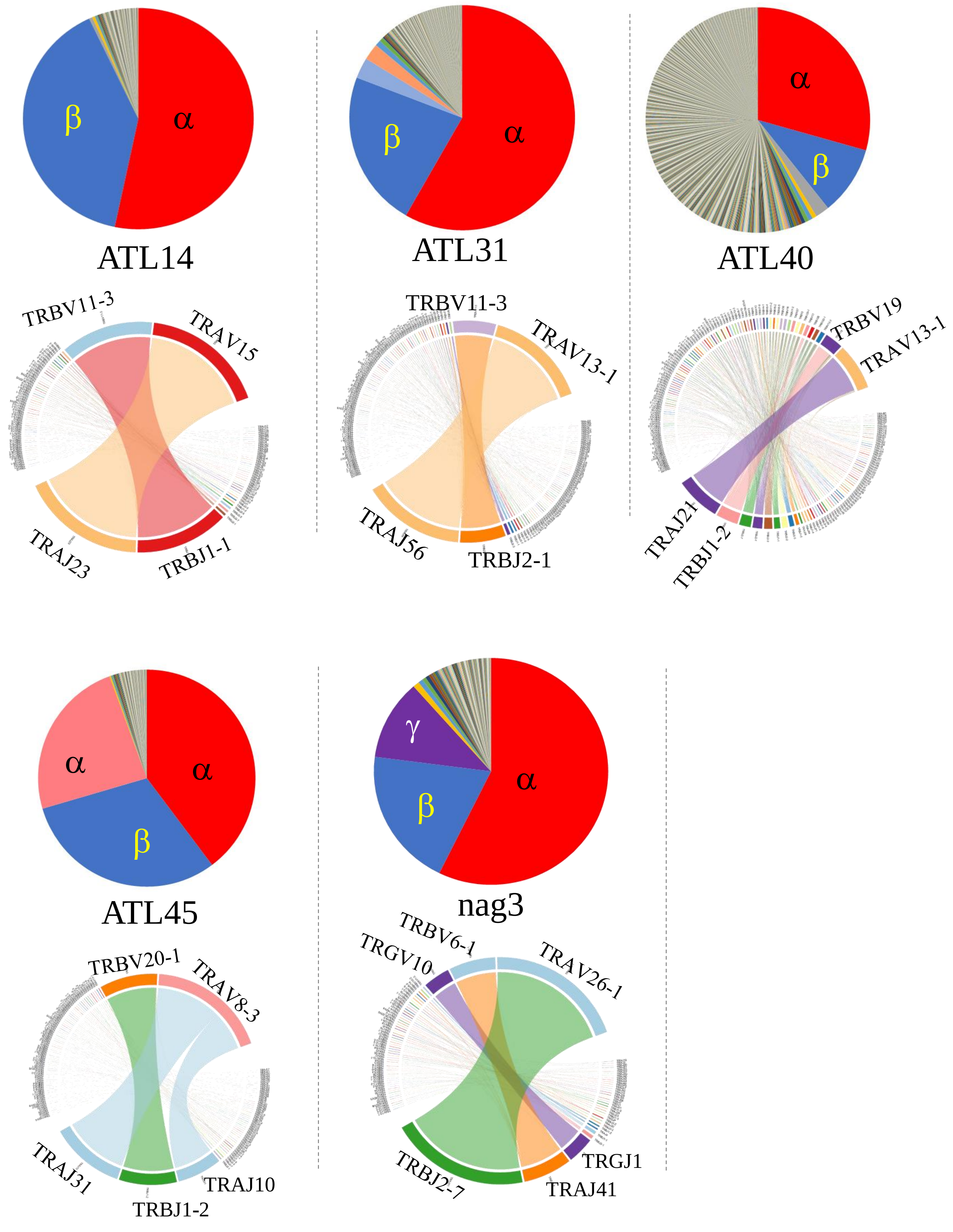

Group#2

Supplementary Figure1-continue

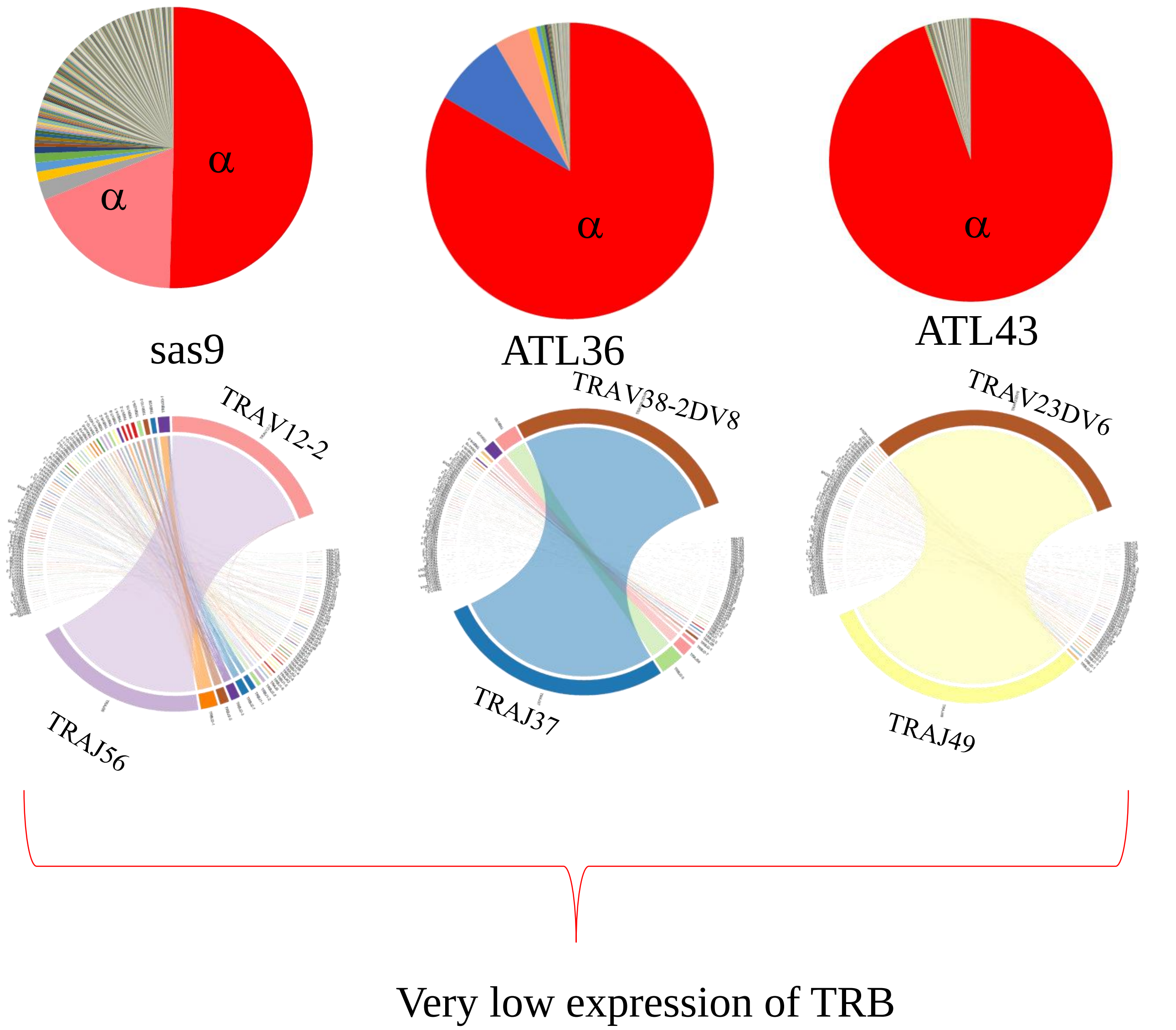

Group#2

Supplementary Figure1-continue

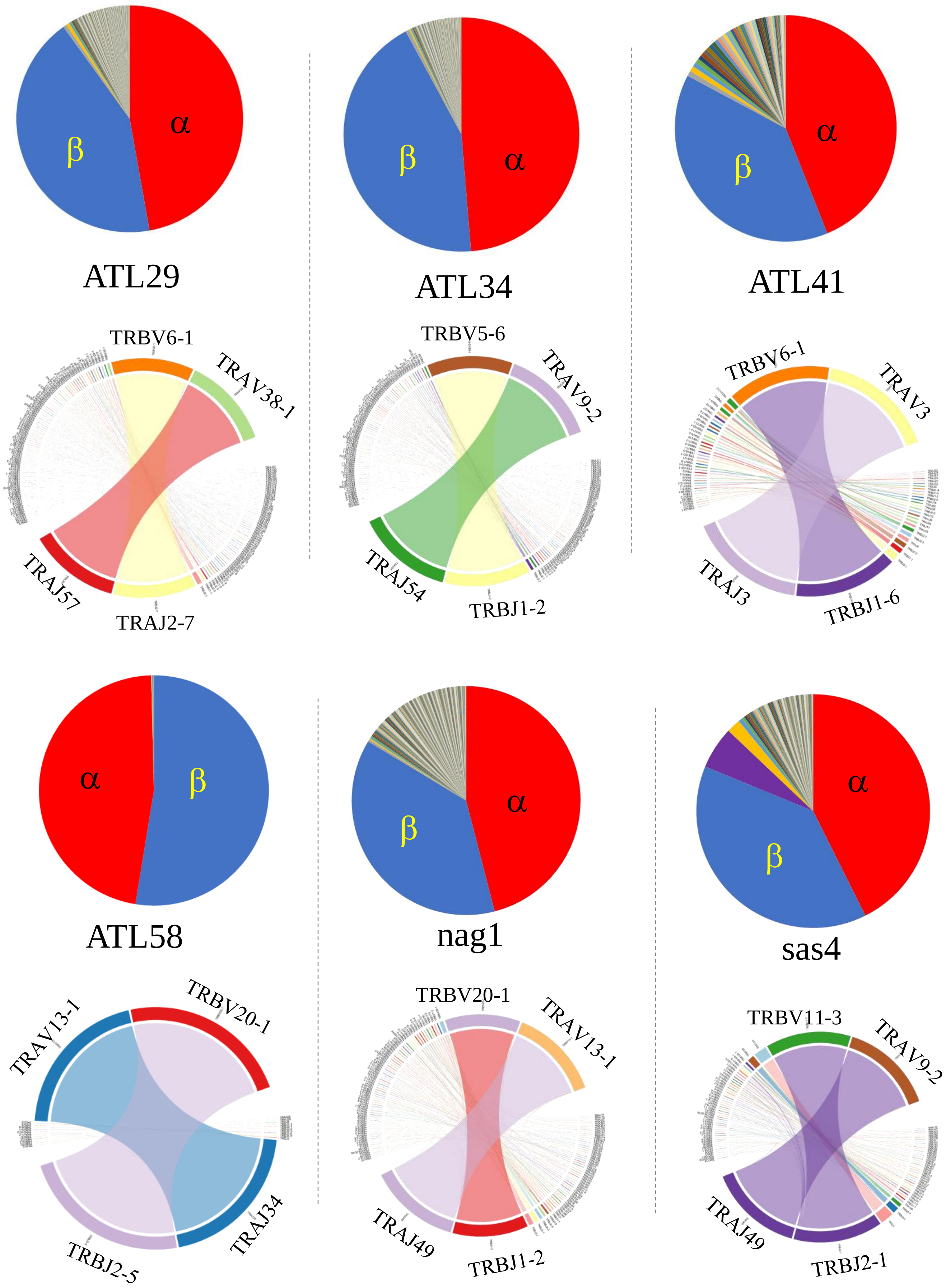

Group#3

Supplementary Figure1-continue

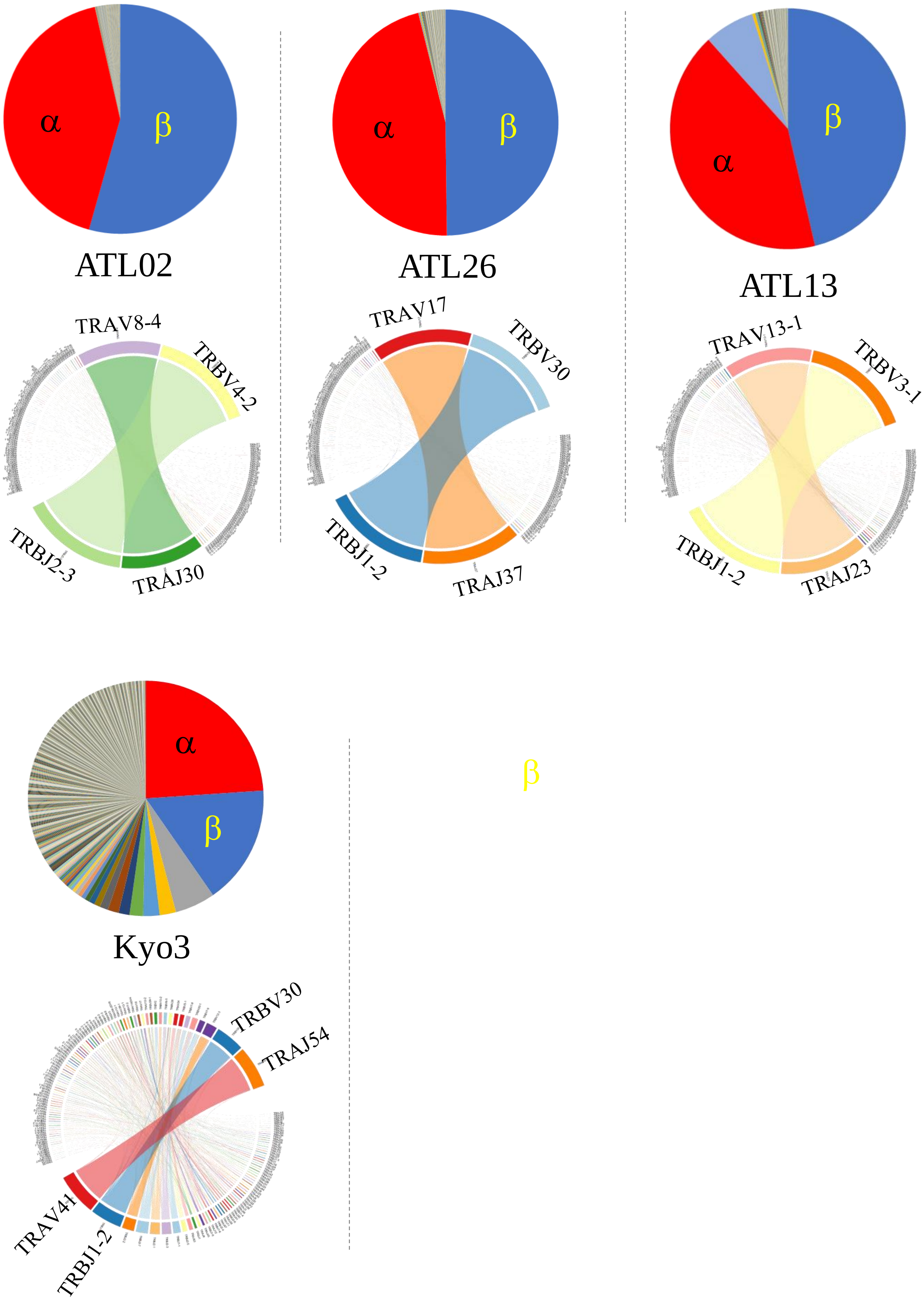

Group#3

Supplementary Figure1-continue

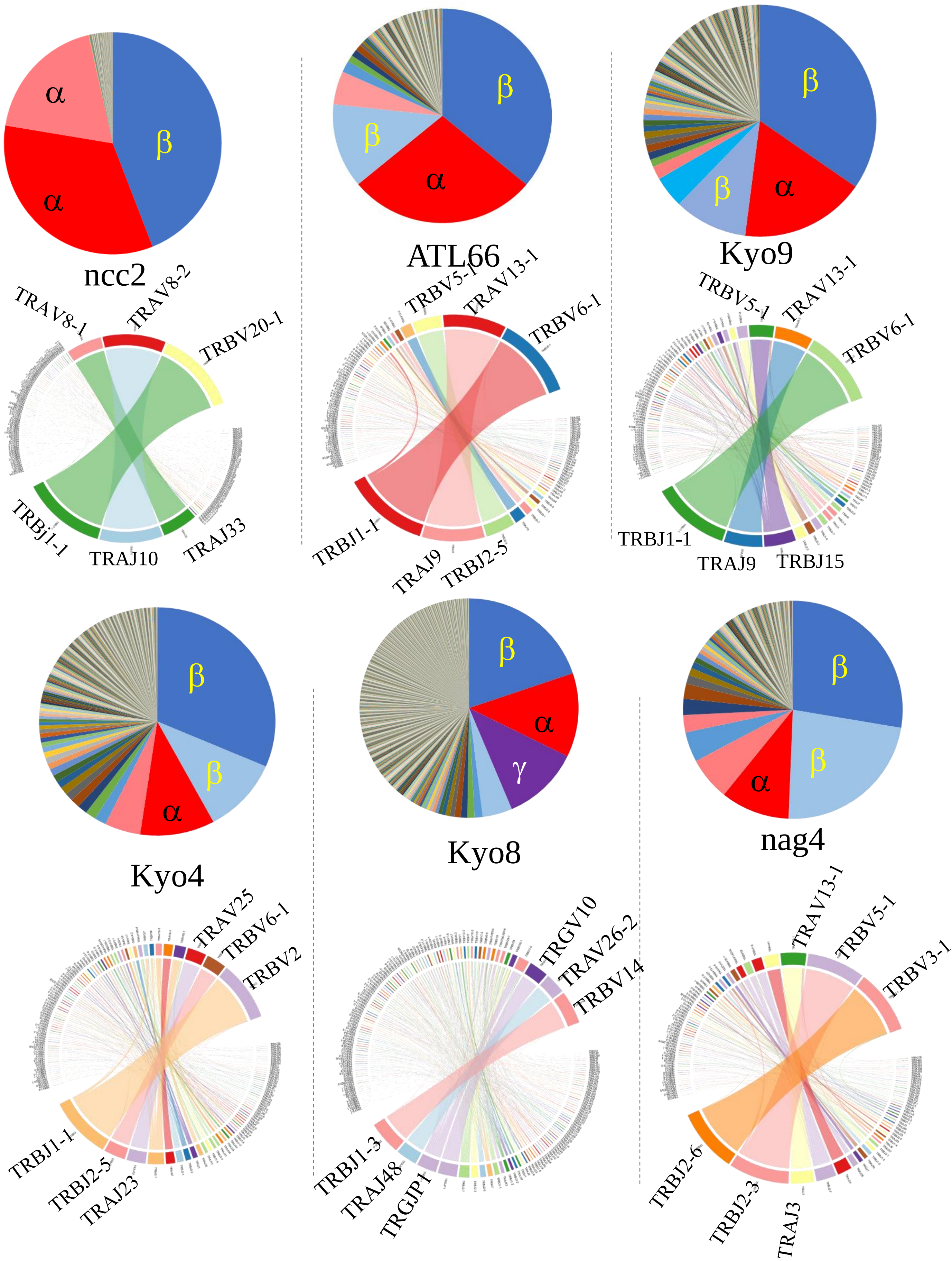

Group#4

Supplementary Figure1-continue

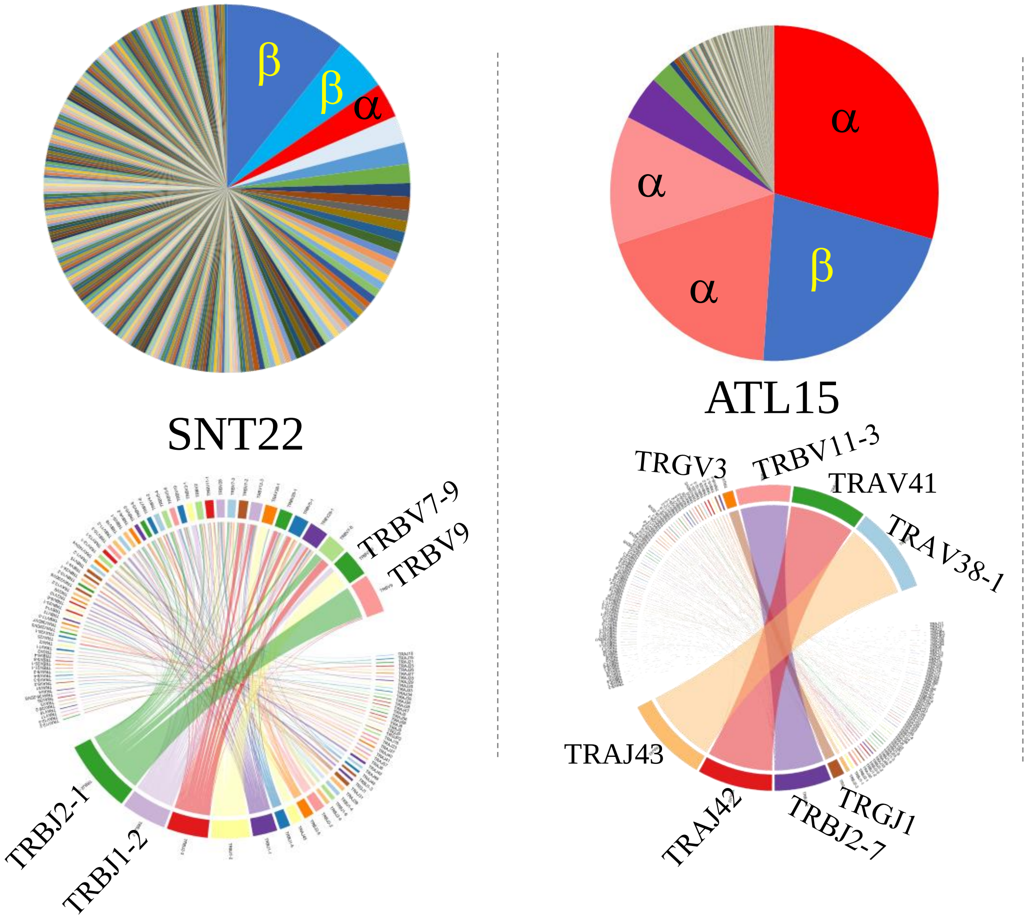

Group#4

Supplementary Figure 1: TCR α and β patterns among all analyzed samples, shown as in Figure 1. Our criteria for classifying samples in four different groups are based on a relative comparison of the expression of TRB and TRA. In a given sample, a dominant clone is a clone of which the largest relative and absolute read counts of TRB or TRA were more than 10% and 100 reads, respectively. We classified these patterns as being either (I) monoclonal, with a single dominant clone, or (II) non-monoclonal. Note that TRB and TRA are expressed concurrently in the same cell, and thus the monoclonal pattern was further divided into three groups: (I)-Group 1: TRB >> TRA; (I)-Group 2: TRB << TRA; (I)-Group 3: TRB ≈ TRA. The non-monoclonal pattern made up Group 4, which contained the rest of the samples.

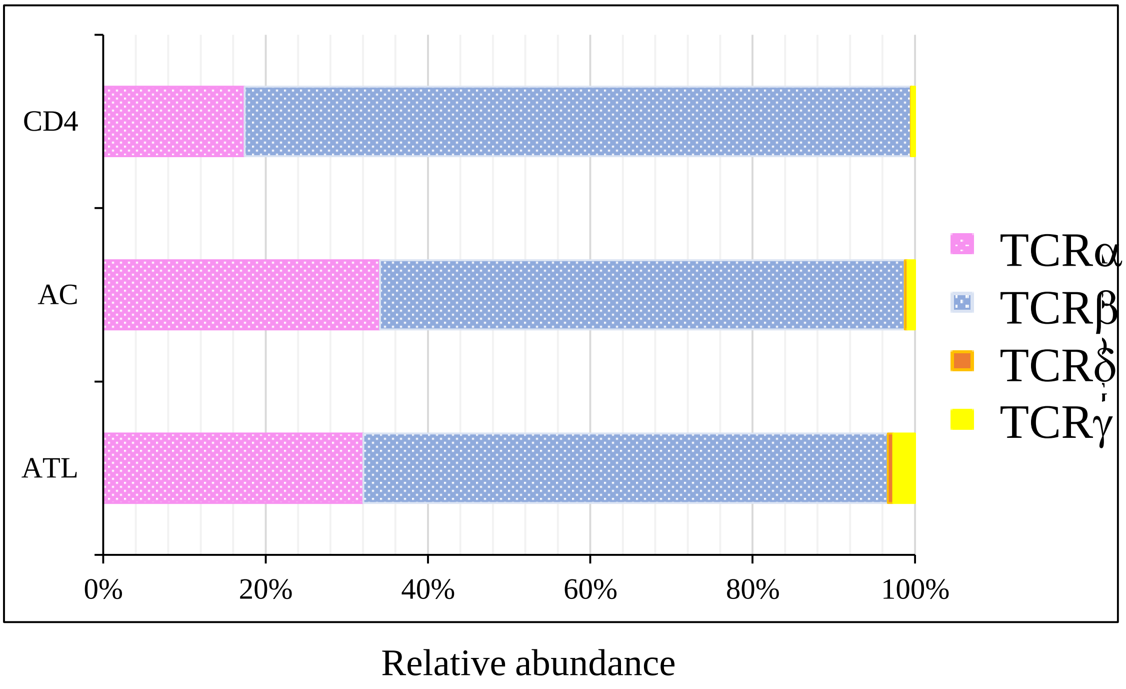

Supplementary Figure 2: Relative abundance of detected TCRα, TCRβ, TCRδ and TCRγ among the samples.

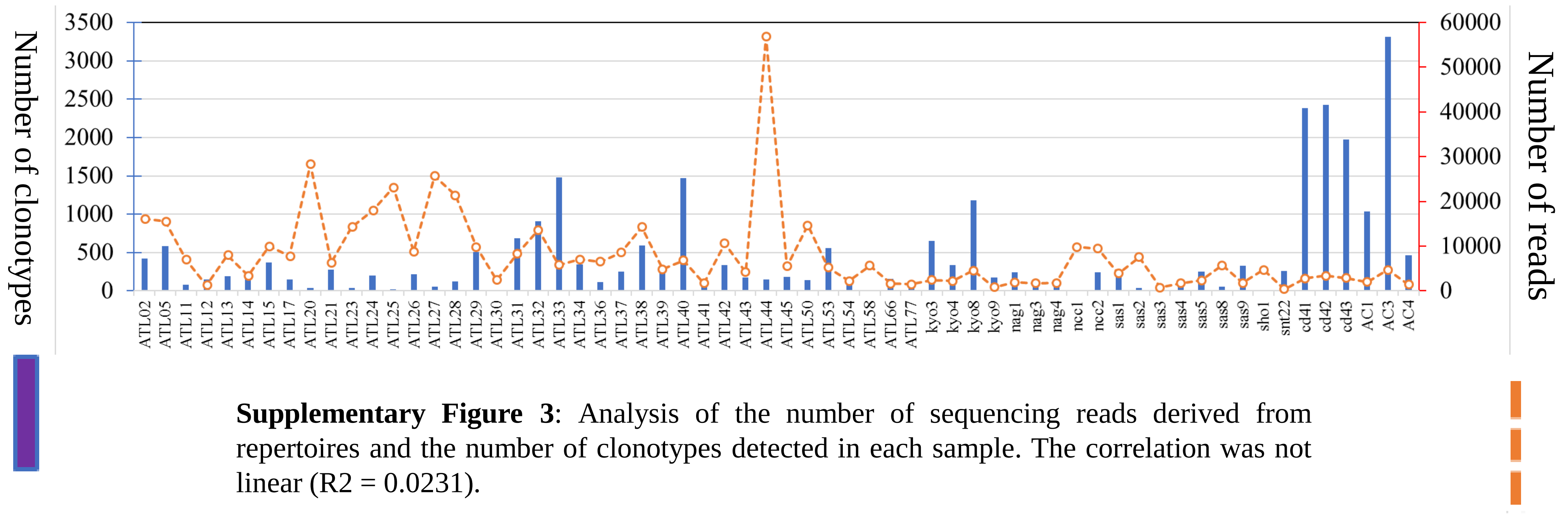

Supplementary Figure 3: Analysis of the number of sequencing reads derived from repertoires and the number of clonotypes detected in each sample. The correlation was not linear (R2 = 0.0231).

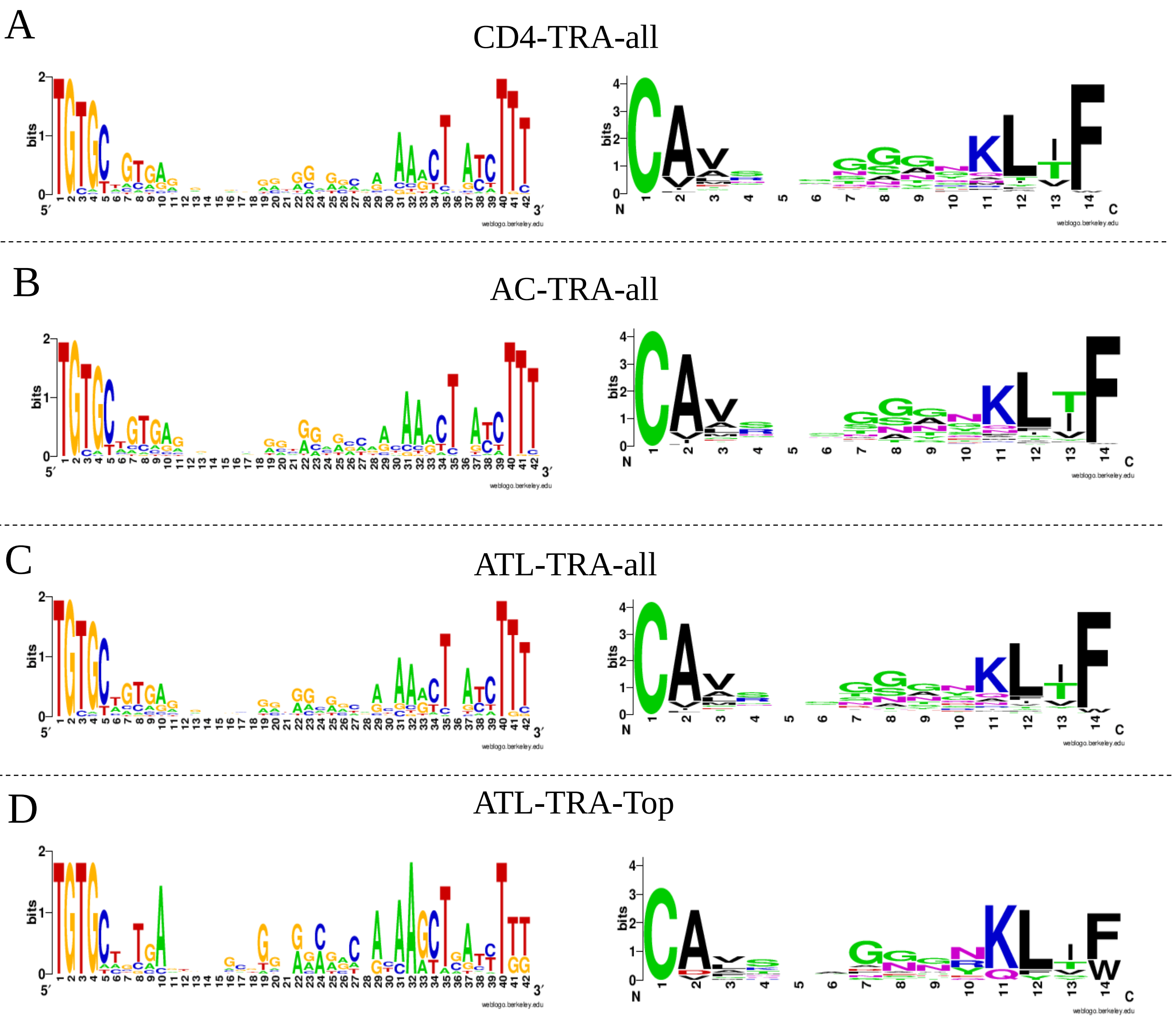

Supplementary Figure 4: TRA gene usage among analyzed samples.

A–C) WebLogo motifs for CDR3 of TRA among all analyzed clonotypes across all samples for (A) the normal controls (CD4), (B) AC samples and (C) ATL samples. D) WebLogo motif for CDR3 of TRA among the top expanded clones with an absolute count of >100 among ATL patients.

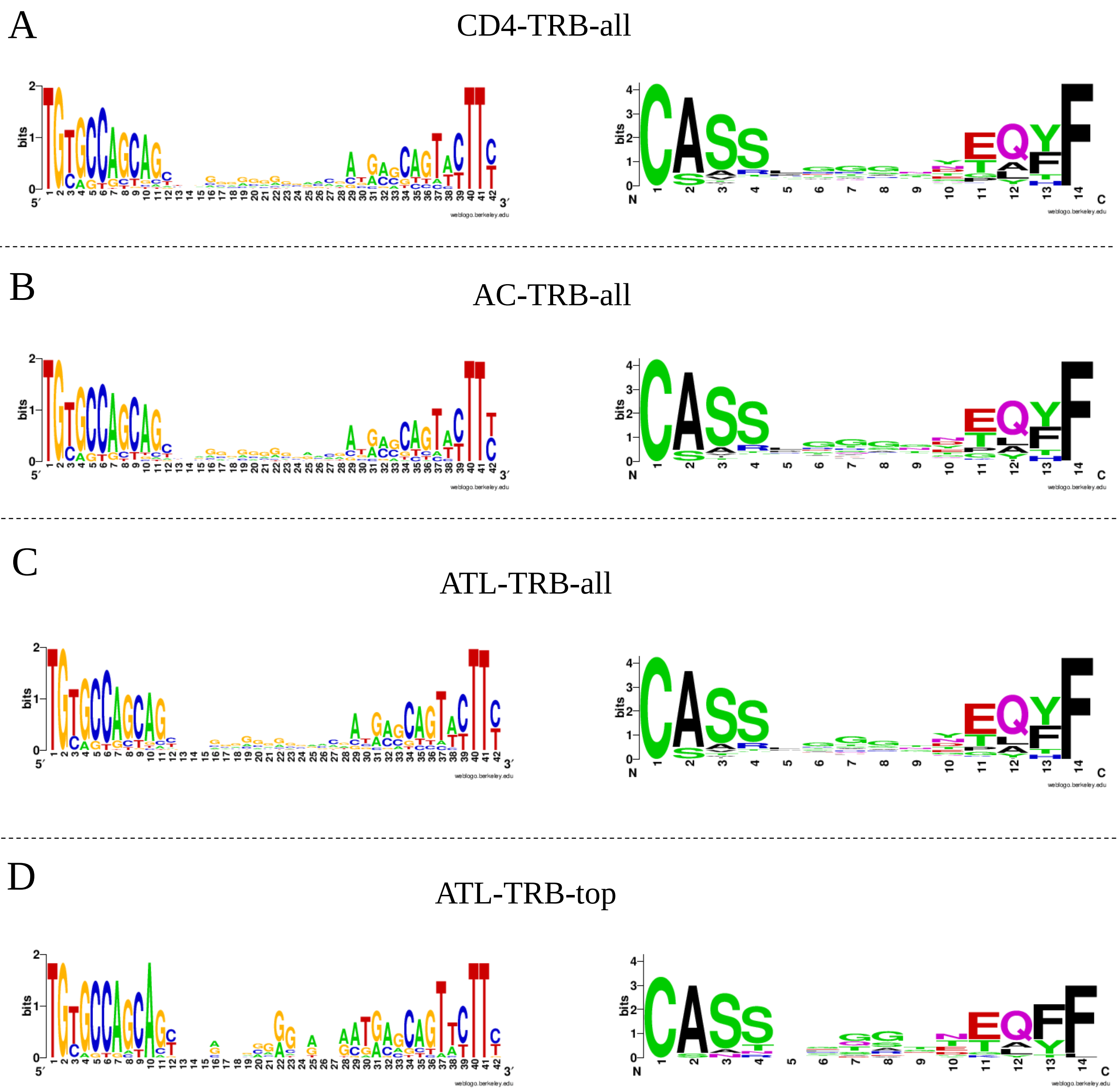

Supplementary Figure 5: TRB gene usage among analyzed samples.

A–C) WebLogo motifs for CDR3 of TRB among all analyzed clonotypes across all samples for (A) the normal controls (CD4), (B) AC samples and (C) ATL samples. D) WebLogo motif for CDR3 of TRB among the top expanded clones with an absolute count of >100 among ATL patients.

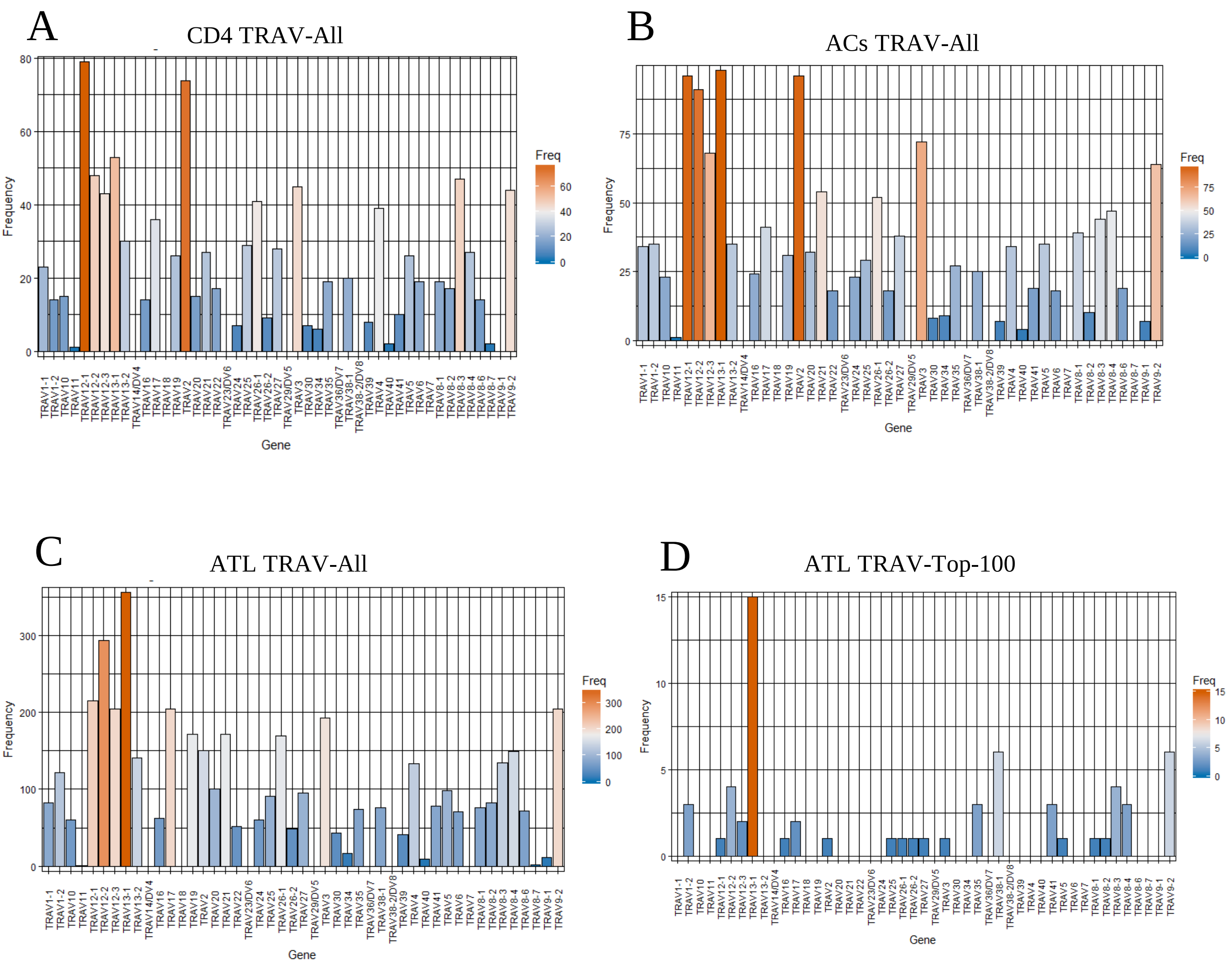

Supplementary Figure 6: Comparing TRAV gene usage among CD4-positive, AC and ATL samples. A) TRAV gene usage among CD4-positive samples. B) TRAV gene usage among AC samples. C) TRAV gene usage among ATL patients. D) TRAV gene usage in the top expanded clones with an absolute count of >100 among ATL patients.

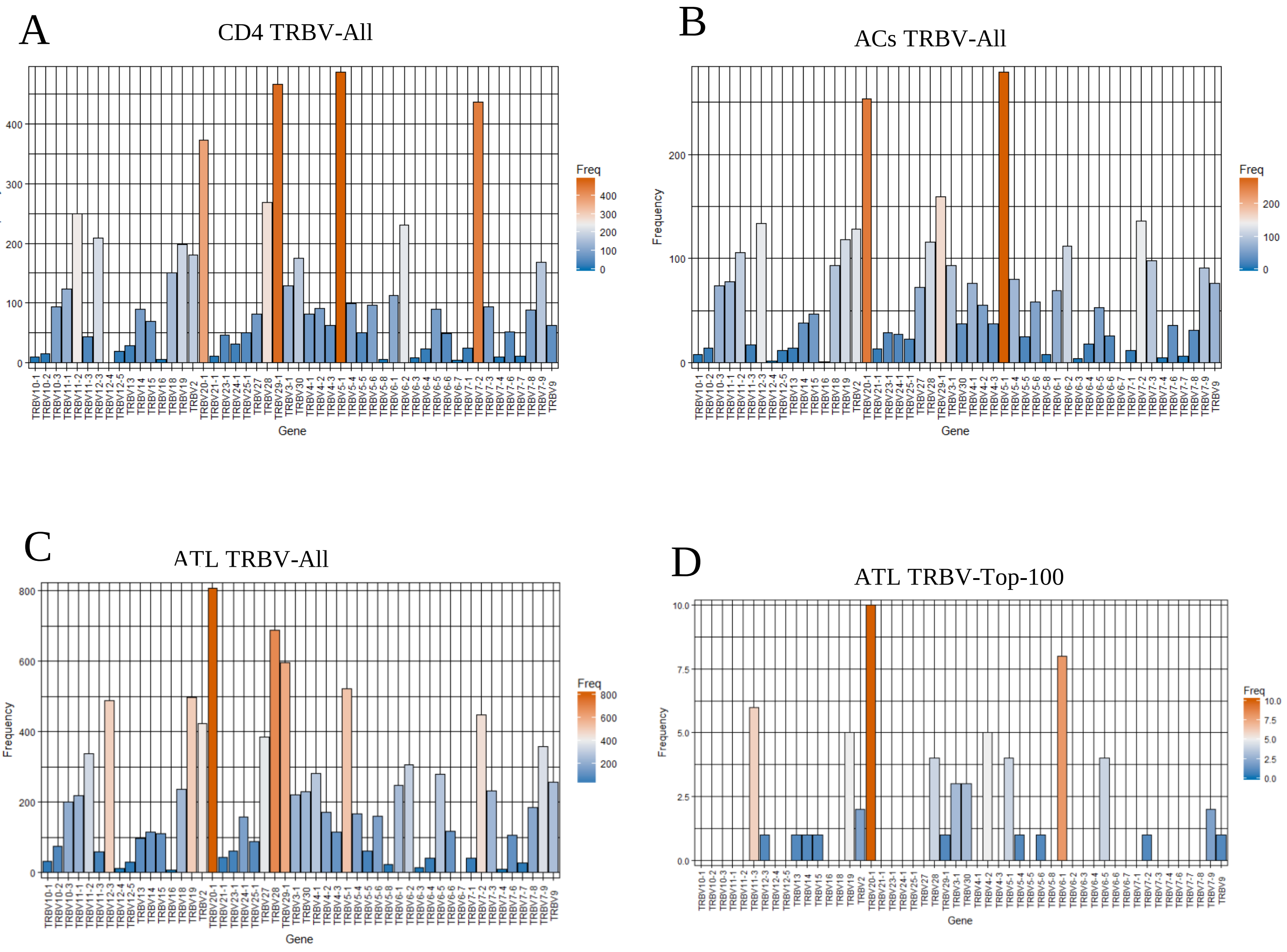

Supplementary Figure 7: Comparing TRBV gene usage among CD4-positive, AC and ATL samples. A) TRBV gene usage among CD4-positive samples. B) TRBV gene usage among AC samples. C) TRBV gene usage among ATL patients. D) TRBV gene usage in the top expanded clones with an absolute count of >100 among ATL patients.

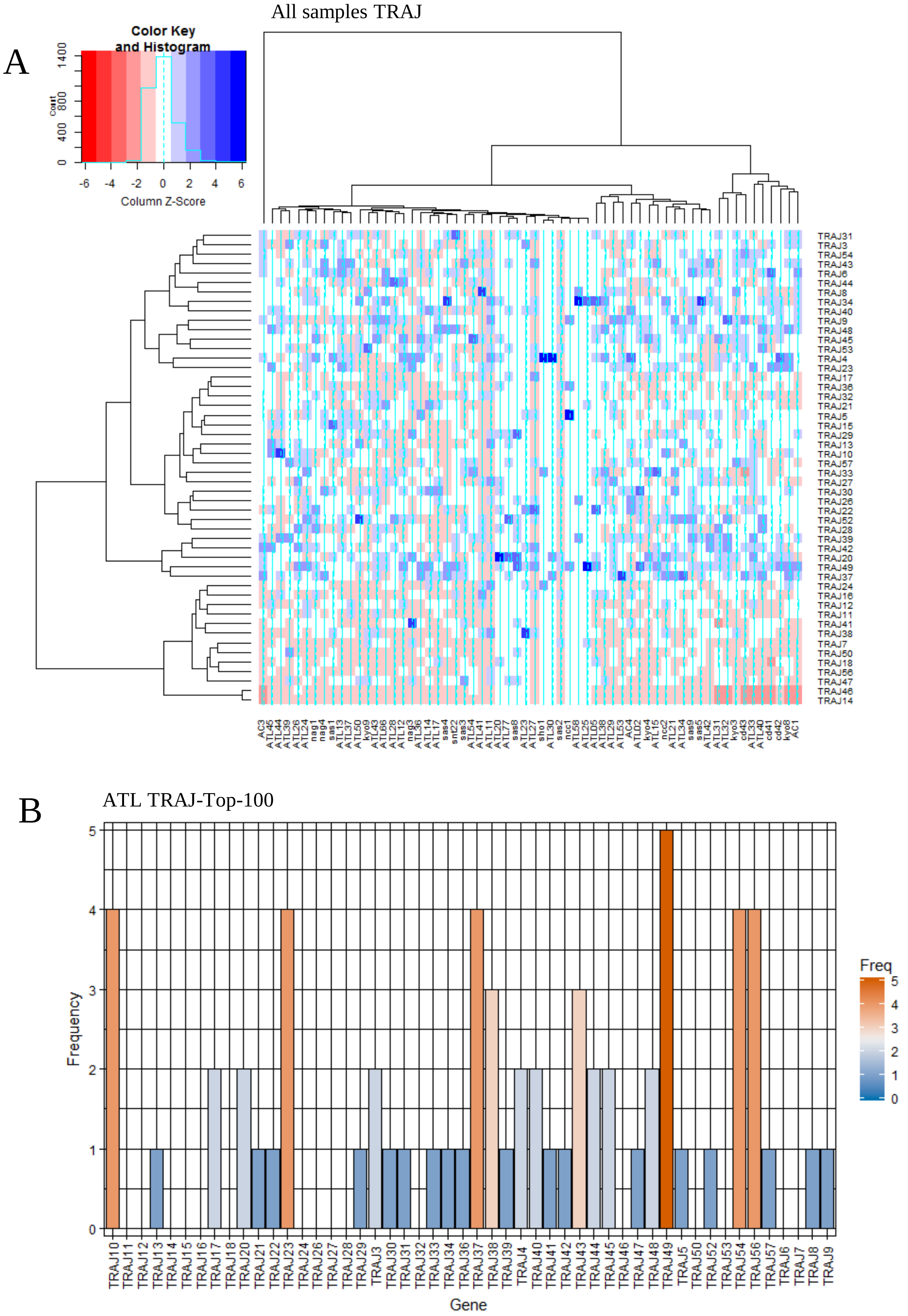

Supplementary Figure 8: Analysis of the T-cell receptor α joining (TRAJ) gene segment usage. A) Histogram of TRAJ gene segment usage across the samples. B) TRAJ gene usage in the top expanded clones with an absolute count of >100 among ATL patients.

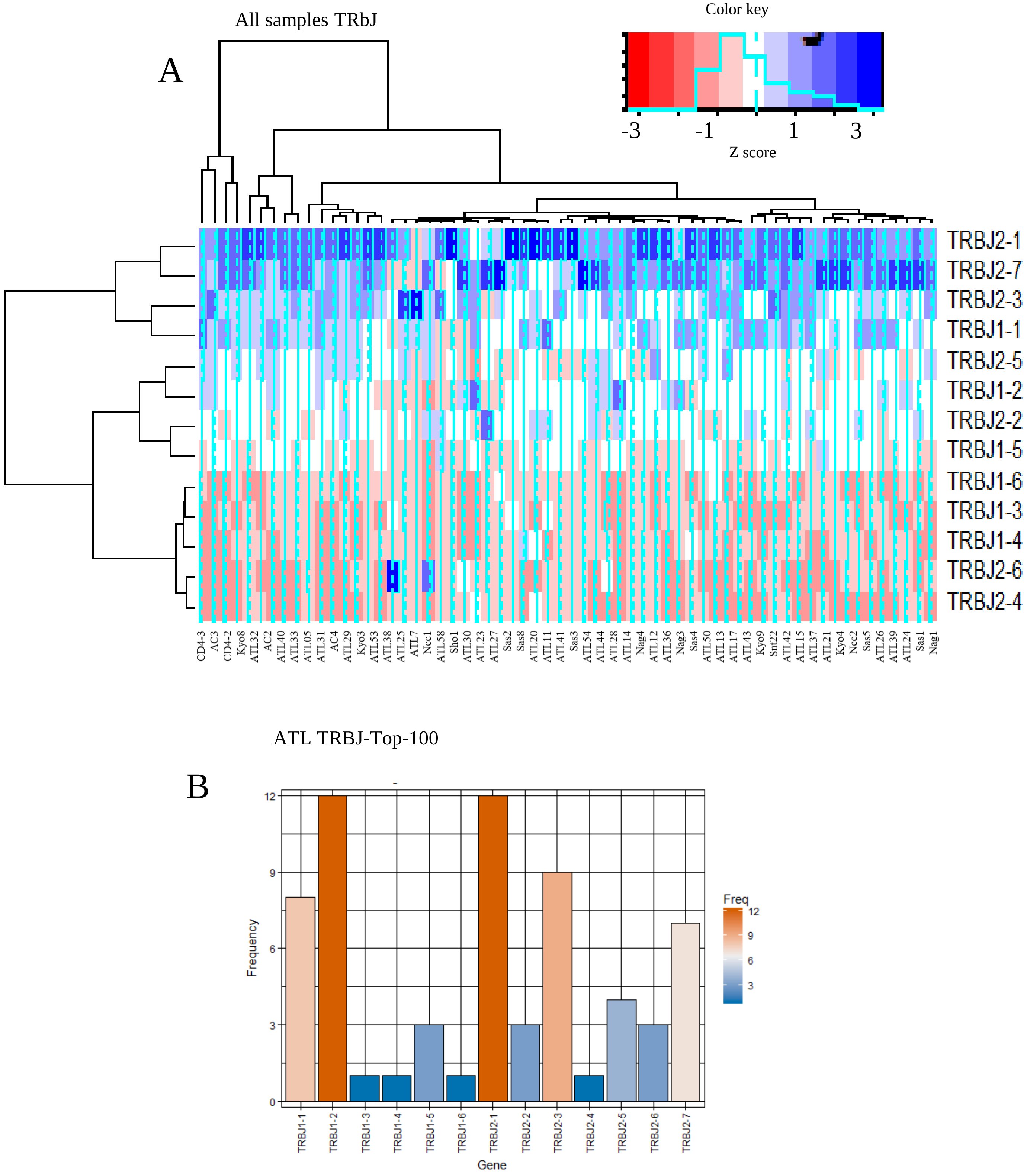

Supplementary Figure 9: Analysis of the T-cell receptor β joining (TRBJ) gene segment usage. A) Histogram of TRAJ gene segment usage across the samples. B) TRAJ gene usage in the top expanded clones with an absolute count of >100 among ATL patients.

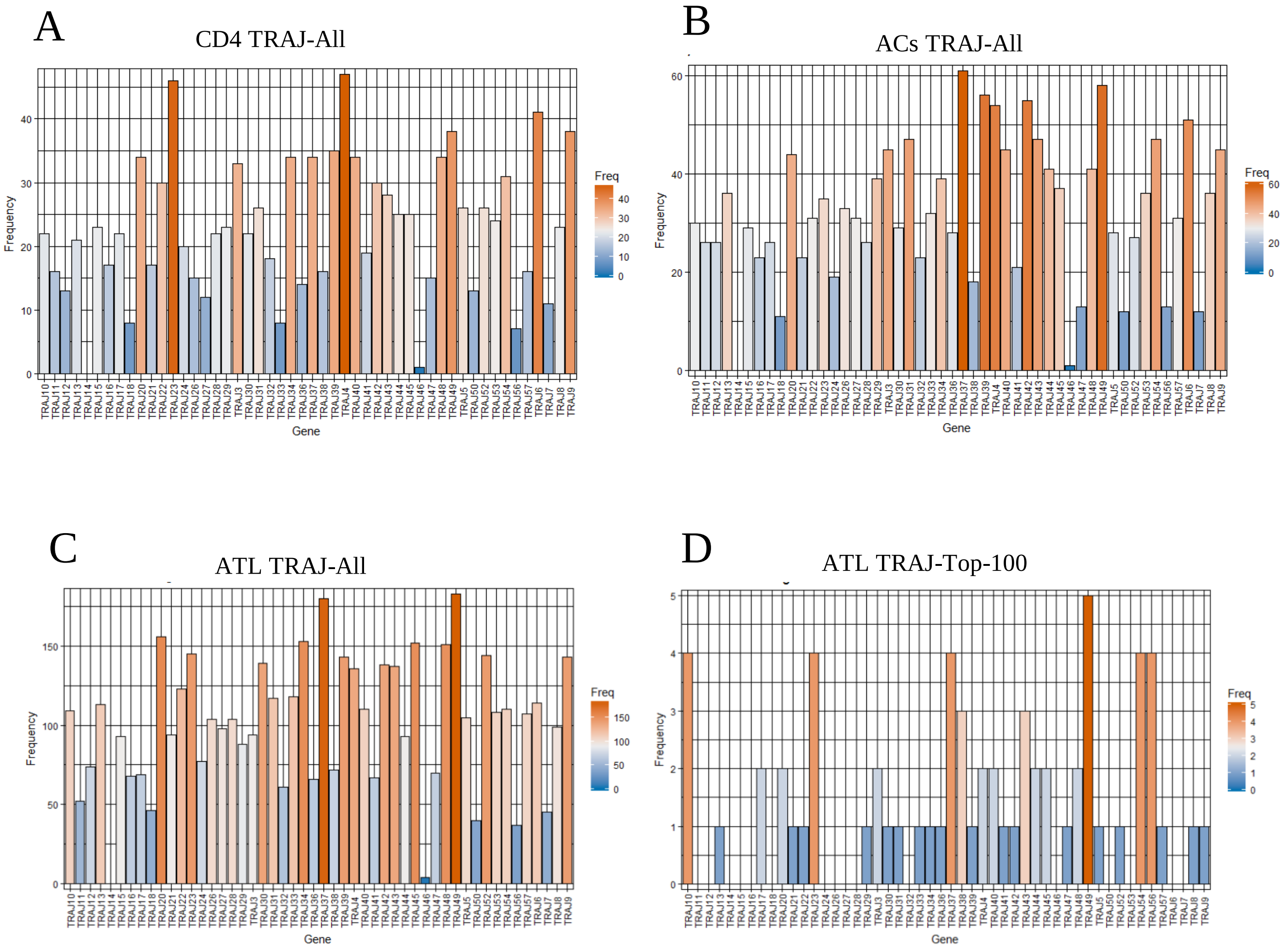

Supplementary Figure 10: TRAJ gene usage in CD4 and AC samples as compared with that in ATL samples.

A) TRAJ gene usage among CD4-positive samples. B) TRAJ gene usage among ACs. C) TRAJ gene usage among ATL patients. D) TRAJ gene usage within the top expanded clones with an absolute count of >100 among ATL patients.

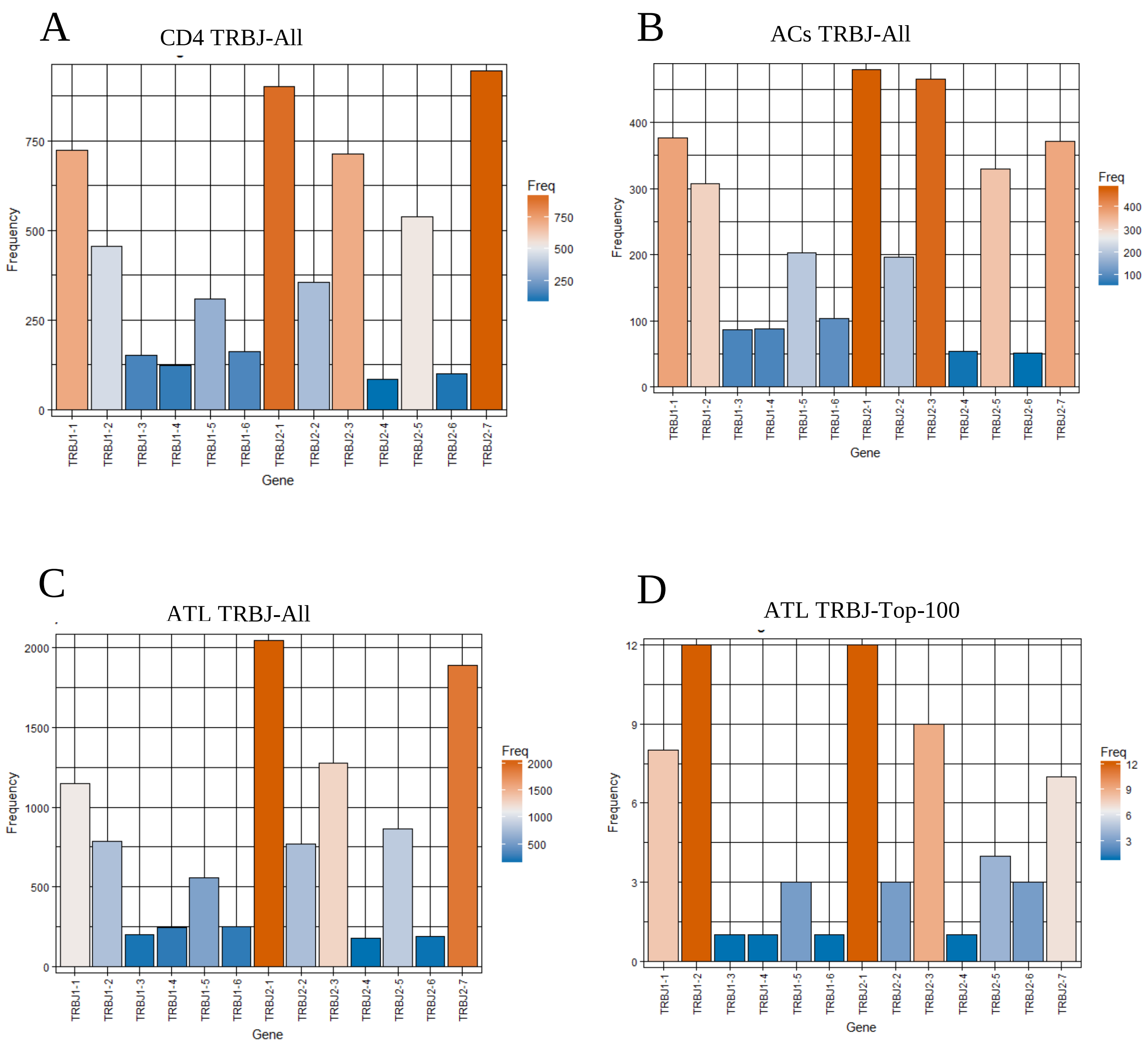

Supplementary Figure 11: TRBJ gene usage in CD4 and AC samples as compared with that in ATL samples. A) TRBJ gene usage among CD4-positive samples. B) TRBJ gene usage among ACs. C) TRBJ gene usage among ATL patients. D) TRBJ gene usage within the top expanded clones with an absolute count of >100 among ATL patients.

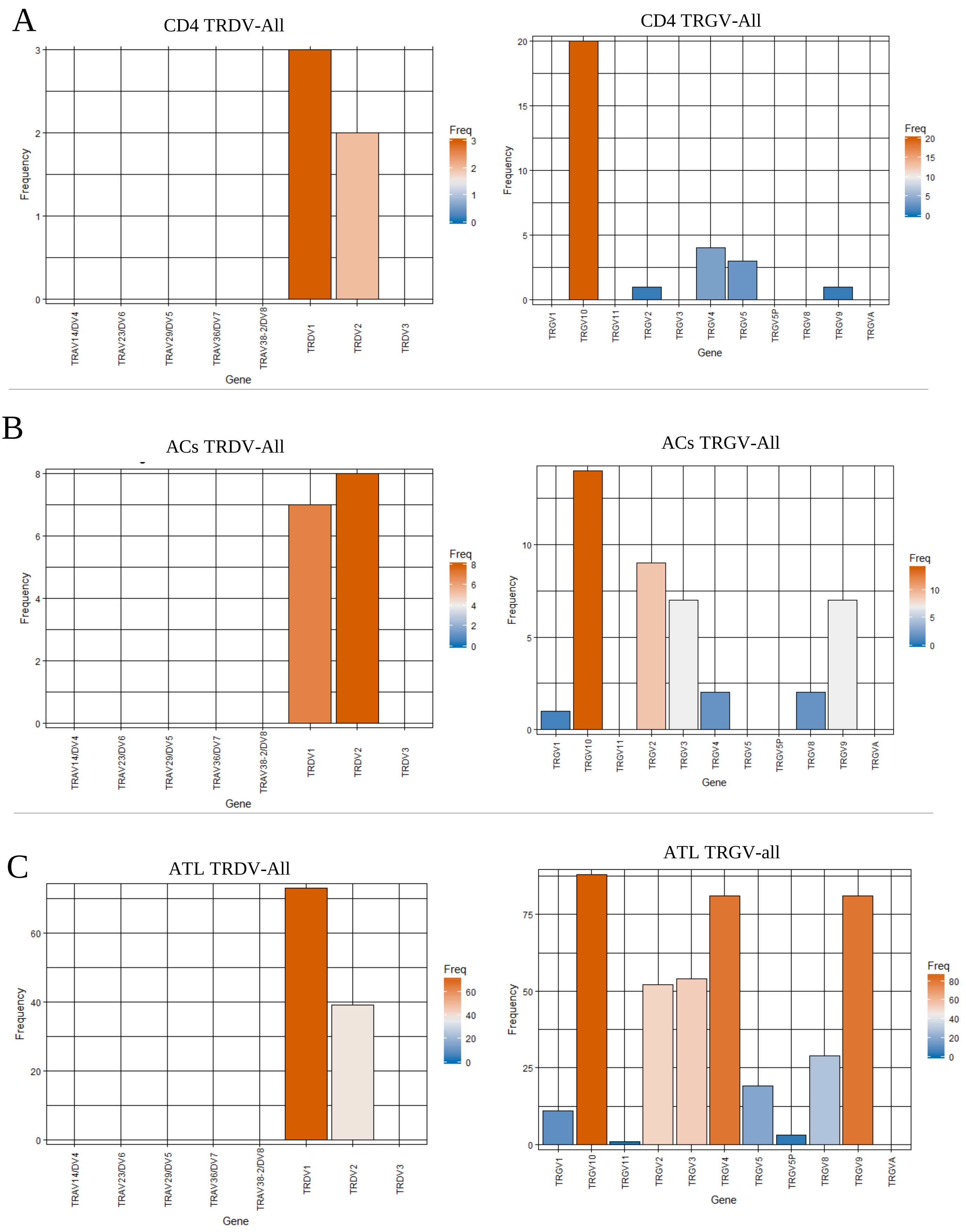

Supplementary Figure 12: Comparing TRDV and TRGV gene usage in CD4 and AC samples as compared with that in ATL samples. A) TRDV and TRGV gene usage in CD4 samples. B) TRDV and TRGV gene usage in ACs. C) TRDV and TRGV gene usage in ATL patients.

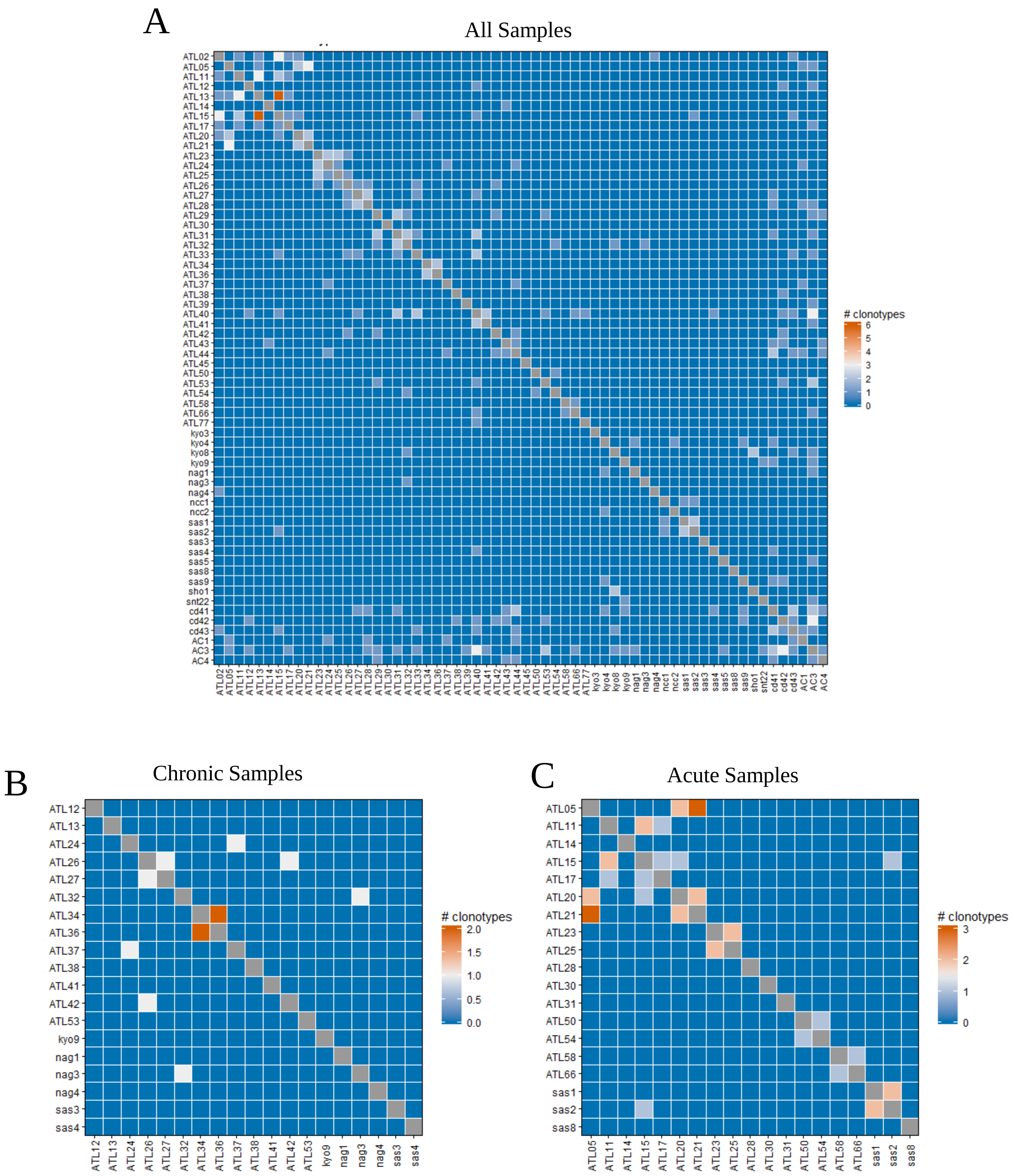

Supplementary Figure 13: Shared clonotypes across samples analyzed at the nucleotide level. A) Shared clonotypes across all samples. B) Shared clonotypes among chronic ATL patients. C) Shared clonotypes among acute ATL patients.

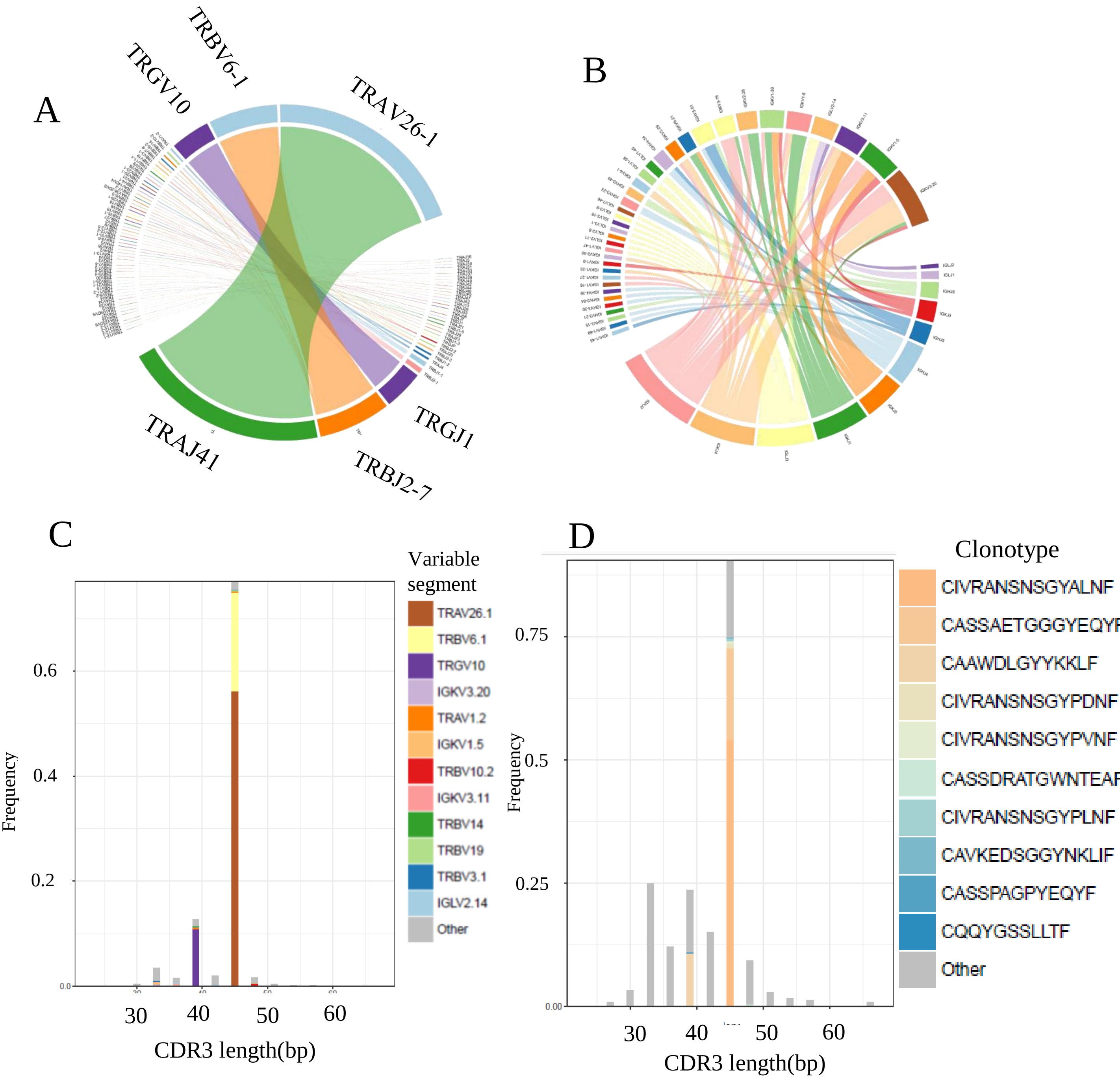

Supplementary Figure 14: Nag-3, an individual with ATL in which TRG expansion had occurred.

A) TCRα rearrangement pattern. B) TCRβ rearrangement pattern. C) Variable gene usage and distribution of CDR3 lengths (in base pairs). D) Amino acid sequences of the clonotypes and distribution of CDR3 lengths (in base pairs).

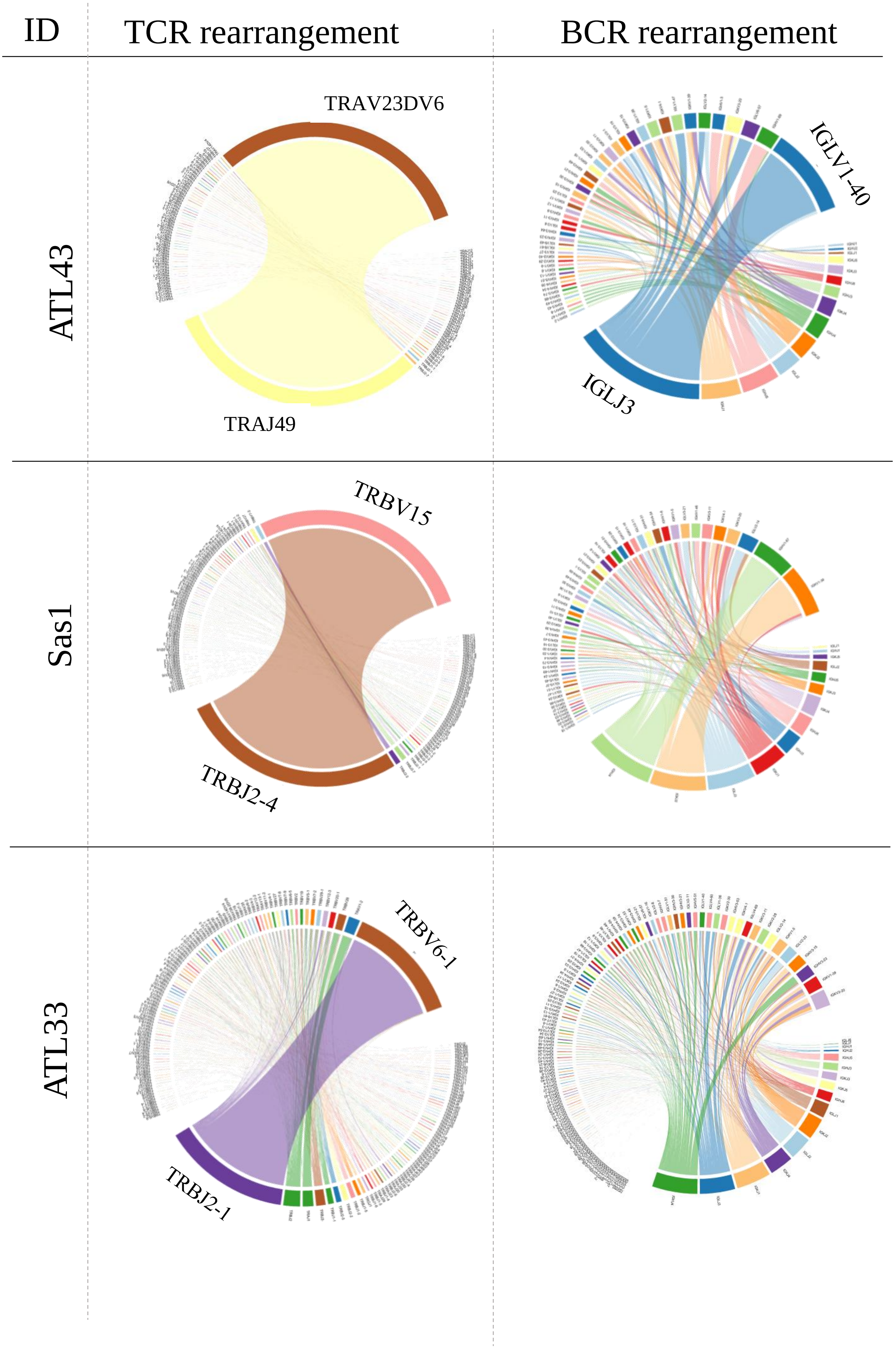

Supplementary Figure 15: Representative examples of BCR patterns among the samples. The circus plots show the rearrangements of the TCR and BCR chains for three representative samples. These samples showed polyclonal BCR rearrangements.

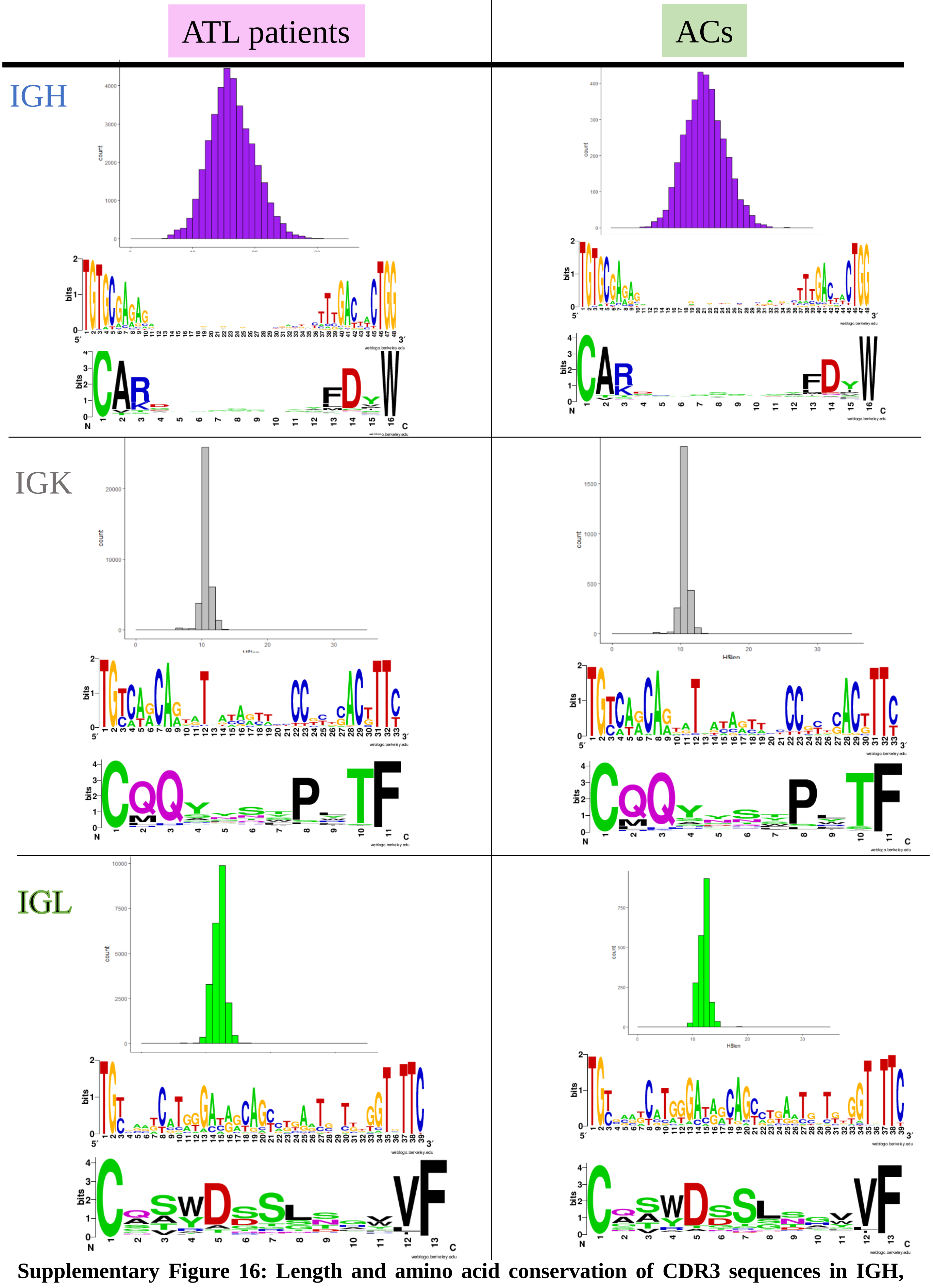

Supplementary Figure 16: Length and amino acid conservation of CDR3 sequences in IGH, IGK and IGL chains across samples.

The length distribution for complete CDR3 calls is shown in the histograms. We selected 16, 11 and 13 amino acid lengths from the CDR3 of IGH, IGK and IGL, respectively, and performed WebLogo analyses. The conservation score is shown on the *y* axis of the logo plots.

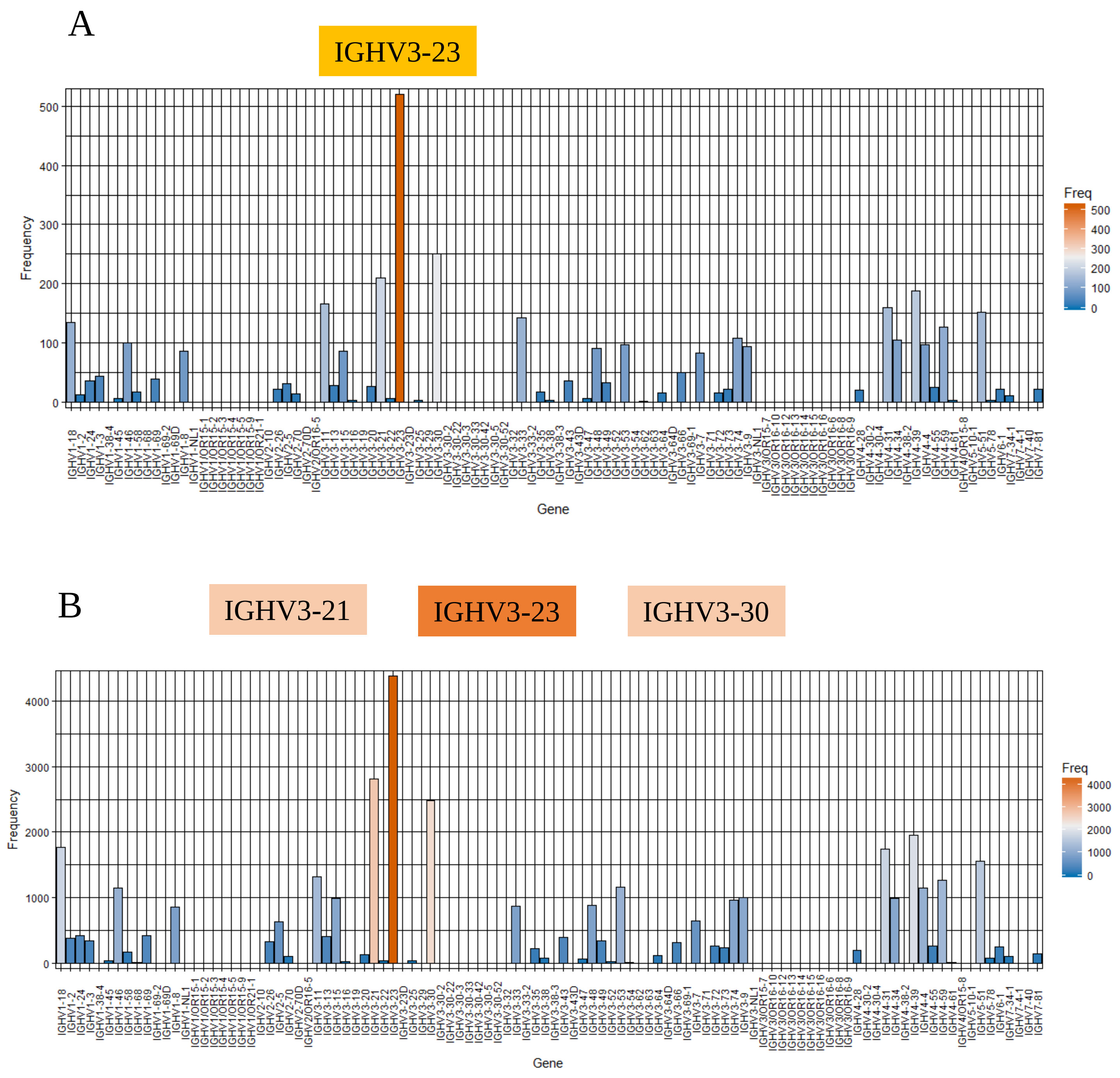

Supplementary Figure 17: IGHV usage in ACs as compared with that in ATL samples. A) IGHV usage among ACs. IGHV3-23 had the highest frequency. B) IGHV usage among ATL patients. In addition to IGHV3-23; IGHV3-21 and IGHV3-30 also had high frequencies.

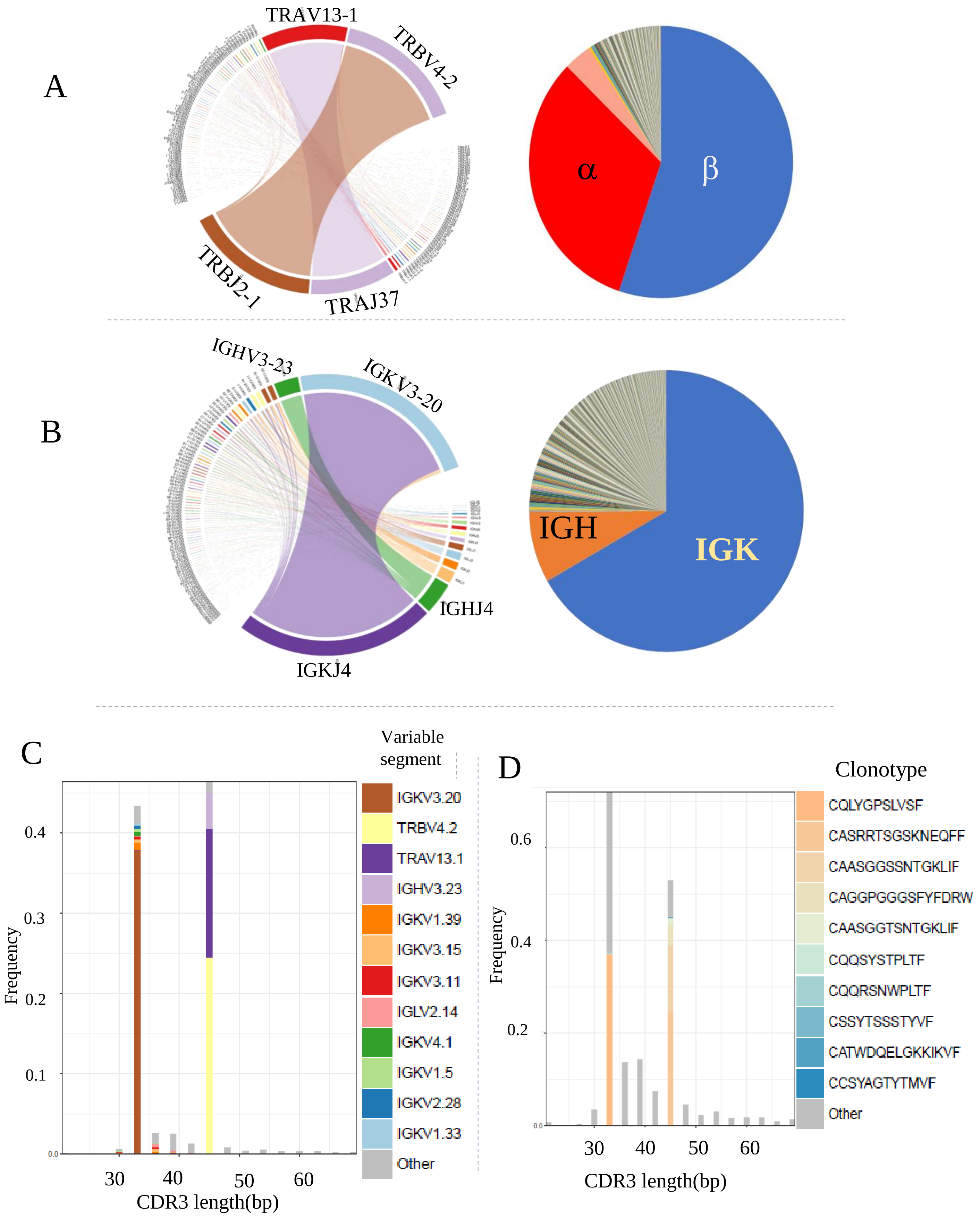

Supplementary Figure 18: ATL21, an individual with ATL in which simultaneous expansion of T cells and B cells had occurred.

A) TCR rearrangement and clonality pattern. B) BCR rearrangement and clonality pattern. C) Variable gene usage and distribution of CDR3 length (in base pairs). D) Amino acid sequence of the clonotypes and the distribution of their CDR3 lengths (in base pairs).

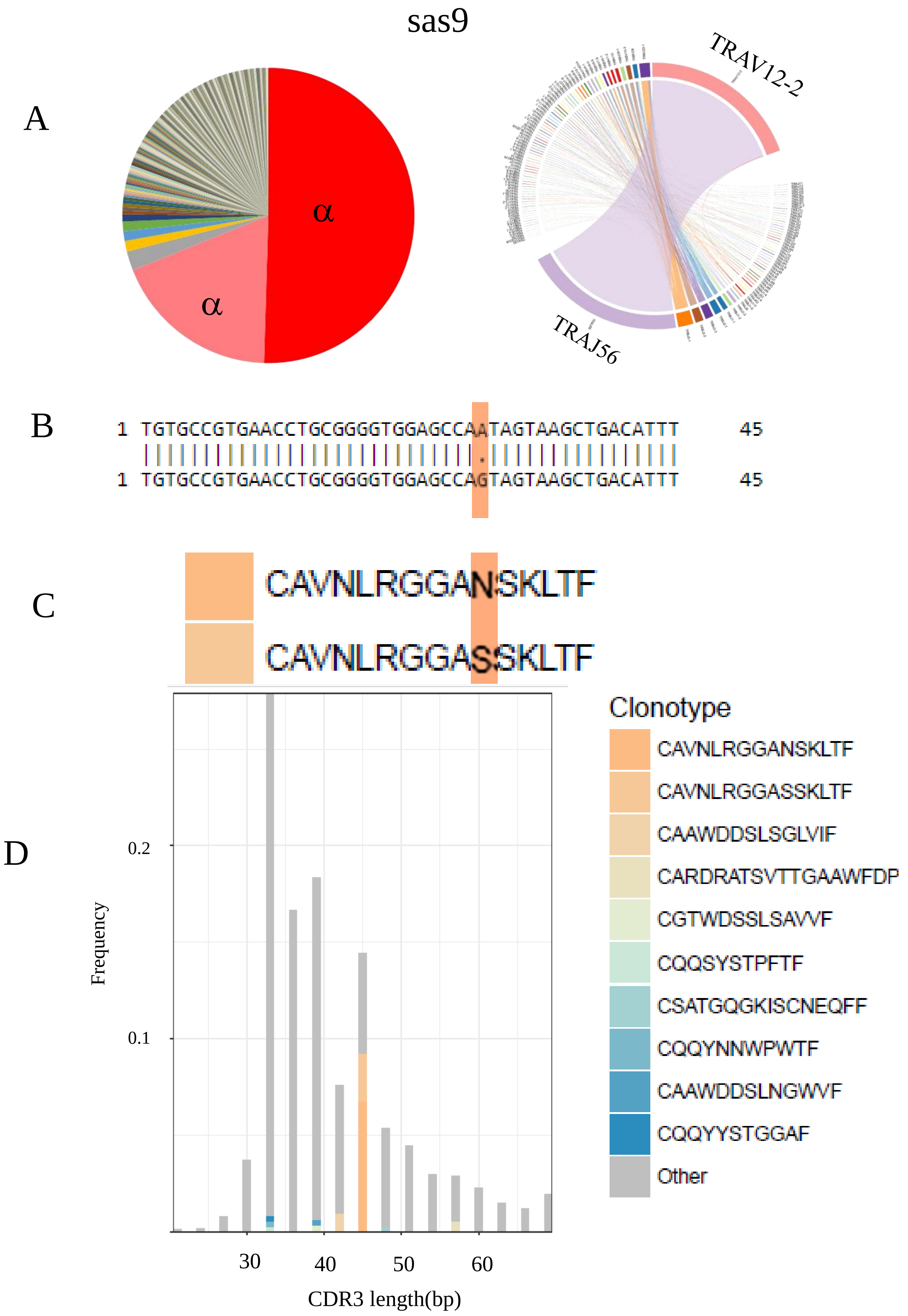

Supplementary Figure 19: Sas9, an individual with ATL in which a non-synonymous mutation in the CDR3 of TCRα had occurred.

A) Clonality pattern and TCR rearrangement. B) Nucleotide sequence of top two sister clones with highlighted point mutation. C) Amino acid sequence of top two sister clones with highlighted amino acid substitution. D) Amino acid sequences of the clonotypes and distribution of their CDR3 lengths (in base pairs).

ATL77

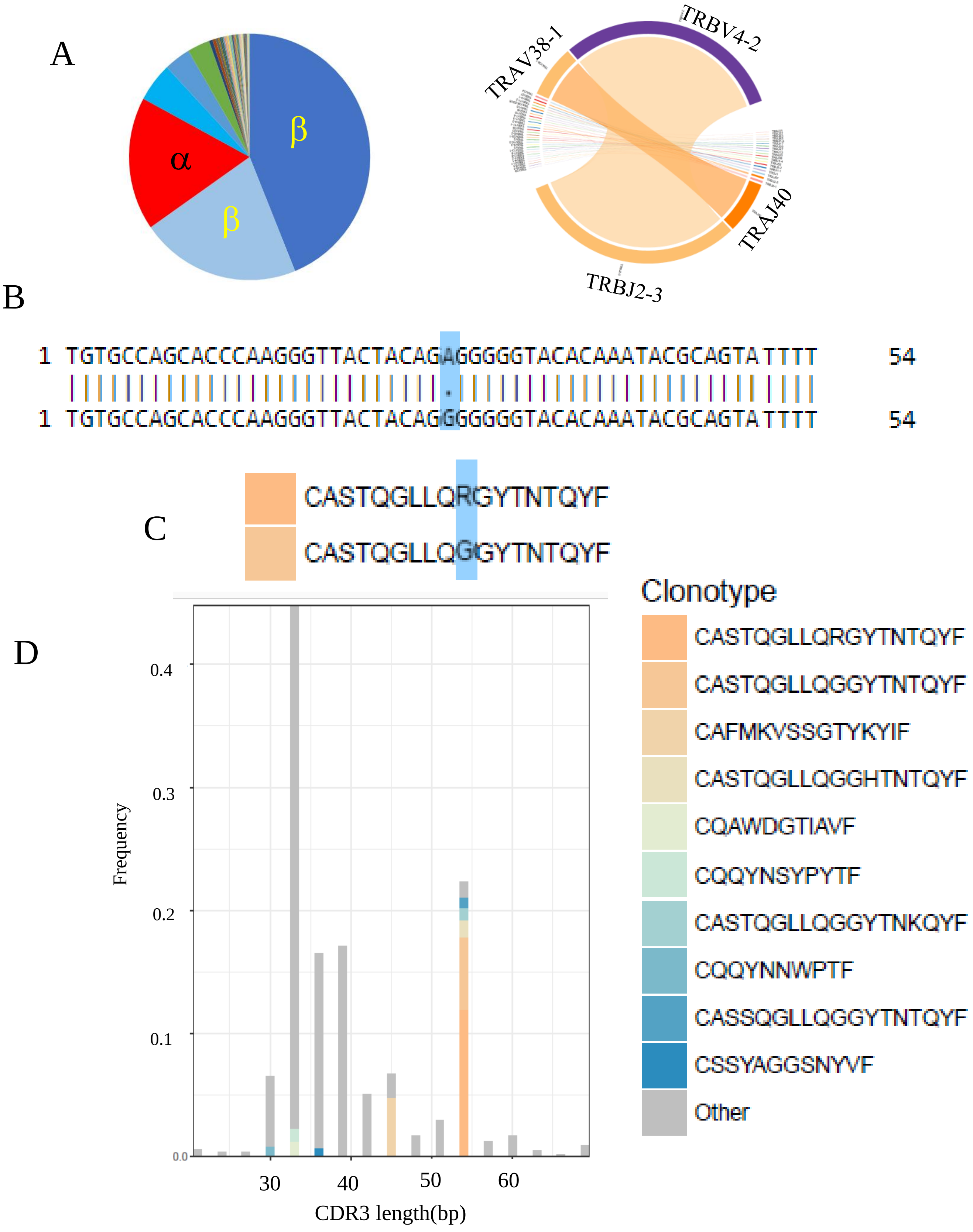

Supplementary Figure 20: ATL77, an individual with ATL in which a non-synonymous mutation in the CDR3 of TCRβ had occurred.

A) Clonality pattern and TCR rearrangement. B) Nucleotide sequence of top two sister clones with highlighted point mutation. C) Amino acid sequence of top two sister clones with highlighted amino acid substitution. D) Amino acid sequences of the clonotypes and distribution of their CDR3 lengths (in base pairs).

Supplementary Table-1:The sample information including ID abbreviations, TRA and TRB rearrangements across analysed samples

sample	gender	age	sub-type	type	PVL	TRA-nu	TRA-AA	TRAV	TRAD	TRAJ	TRA-len	TRB-nu	TRB-AA	TRBV	TRBD	TRBJ	TRB-len	clonotypes	reads*	Gini-Simpson
ATL05	M	60	Acute	aggressive	47.296	TGTGCTC	CALKNQG	TRAV9-2	.	TRAJ23	12	TGTGCCA	CASRQDP	TRBV4-2	TRBD2	TRBJ2-3	17	583	15545	0.5333584
ATL11	F	72	Acute	aggressive	141.74	TGTGCTT	CAFMDAG	TRAV38-1	.	TRAJ38	14	TGTGCCA	CASSLNQ	TRBV11-3	.	TRBJ1-1	13	81	6948	0.4667009
ATL14	F	60	Acute	aggressive	114.13	TGTGCAG	CAESSQG	TRAV5	.	TRAJ23	12	TGTGCCA	CASSTGQ	TRBV11-3	TRBD1	TRBJ1-1	13	148	3395	0.5583514
ATL15	M	77	Acute	aggressive	84.923	TGTGCTG	CAVRPLR	TRAV41	.	TRAJ42	15	TGTGCCA	CASSLDGS	TRBV11-3	.	TRBJ2-7	13	373	9989	0.8125711
ATL17	M	59	Acute	aggressive	206.75	TGTGCAG	CAASIKA	TRAV13-1	.	TRAJ17	14	TGCAGCG	CSVEGQG	TRBV29-1	TRBD1	TRBJ2-6	16	145	7782	0.3338035
ATL20	M	64	Acute	aggressive	83.176	TGTGCAG	CAASNDY	TRAV13-1	.	TRAJ20	11	TGTGCCA	CASSDYN	TRBV7-9	.	TRBJ2-1	11	38	28292	0.4781733
ATL21	F	76	Acute	aggressive	26.997	TGTGCAG	CAASGGS	TRAV13-1	.	TRAJ37	15	TGTGCCA	CASRRTS	TRBV4-2	TRBD2	TRBJ2-1	15	280	6338	0.5905581
ATL23	F	73	Acute	aggressive	88.747	TGTGACG	CDVTGAG	TRAV12-2	.	TRAJ38	14	TGTGCCA	CASSYTG	TRBV6-5	TRBD1	TRBJ1-2	13	40	14271	0.4466035
ATL25	M	71	Acute	aggressive	74.14	TCTGCTG	SAGQLGT	TRAV35	.	TRAJ49	13	TGTGCCA	CASSKFGI	TRBV19	.	TRBJ2-6	17	23	23093	0.3010963
ATL28	M	45	Acute	aggressive	81.904	TGTGCAG	CAGDGGT	TRAV27	.	TRAJ44	13	TGTGCCA	CASSSLTG	TRBV6-5	TRBD1	TRBJ1-2	14	123	21359	0.4873502
ATL30	M	80	Acute	aggressive	57.342	TGTGCTG	CAVFSGG	TRAV1-2	.	TRAJ4	13	TGTGCCA	CASGSTG	TRBV2	TRBD1	TRBJ2-1	13	14	2457	0.322673
ATL31	M	69	Acute	aggressive	64.616	TGTGCAG	CAARGAN	TRAV13-1	.	TRAJ56	12	TGTGCCA	CASSPRGS	TRBV11-3	TRBD2	TRBJ2-1	14	685	8340	0.6078842
ATL44	M	45	Acute	aggressive	NA	TGTGCAA	CAMSEGG	TRAV12-3	.	TRAJ10	11	TGTGCCA	CANTRTS	TRBV28	TRBD2	TRBJ2-1	14	149	56833	0.3399101
ATL45	F	69	Acute	aggressive	NA	TGTGCTG	CAVGAQN	TRAV8-3	.	TRAJ31	12	TGCAGTG	CSASAGIN	TRBV20-1	TRBD1	TRBJ1-2	13	186	5485	0.6902375
ATL50	F	71	Acute	aggressive	107.12	TGTGCTC	CALSNVA	TRAV9-2	.	TRAJ52	17	TGTGCCA	CASRTVD	TRBV5-4	TRBD1	TRBJ2-1	14	137	14679	0.3030884
ATL54	M	61	Acute	aggressive	36.616	TGTGCTC	CALSDAG	TRAV9-2	.	TRAJ38	14	TGTGCCA	CASSIGQG	TRBV19	TRBD1	TRBJ2-3	14	134	2201	0.5826739
ATL58	F	64	Acute	aggressive	76.443	TGTGCAG	CAASNDT	TRAV13-1	.	TRAJ34	12	TGCAGTG	CSASGSR	TRBV20-1	TRBD2	TRBJ2-5	12	15	5637	0.502352
ATL77	M	86	Acute	aggressive	NA	TGTGCTT	CAFMKVS	TRAV38-1	.	TRAJ40	15	TGTGCCA	CASTQGL	TRBV4-2	TRBD1	TRBJ2-3	14	42	1462	0.7260616
NCC2	M	61	Acute	aggressive	NA	TGTGTTG	CVVSDPV	TRAV8-2	.	TRAJ10	16	TGCAGTG	CSARMNT	TRBV20-1	.	TRBJ1-1	11	245	9460	0.6581036
Sas1	M	77	Acute	aggressive	84.696	TGTGCAA	CAMRESD	TRAV14D	.	TRAJ34	14	TGTGCCA	CATSRLIP	TRBV15	TRBD2	TRBJ2-4	22	219	3875	0.2106917
Sas2	M	78	Acute	aggressive	46.487	TGTGCAG	CAASKTG	TRAV13-1	.	TRAJ36	13	TGTGCCA	CASSLQD	TRBV19	TRBD1	TRBJ2-2	13	41	7549	0.4034936
Sas8	M	73	Acute	aggressive	NA	TGTGCAA	CAIRNSG	TRAV12-3	.	TRAJ29	13	TGTGCCA	CASSVAG	TRBV9	TRBD1	TRBJ2-7	12	52	5699	0.4912837
ATL12	M	59	Chronic	Indolent	67.946	TGTGCTC	CALTRAG	TRAV16	.	TRAJ10	14	TGTGCCA	CASSEGG	TRBV6-1	TRBD1	TRBJ1-2	12	149	1253	0.6346091
ATL13	M	58	Chronic	Indolent	71.404	TGTGCAG	CAANQG	TRAV13-1	.	TRAJ23	11	TGTGCCA	CASSPTG	TRBV3-1	TRBD1	TRBJ1-2	13	189	7989	0.605039
ATL24	M	63	Chronic	Indolent	114.24	TGTGCTG	CAVSGSA	TRAV8-4	.	TRAJ22	12	TGTGCCA	CASSYGT	TRBV6-5	TRBD1	TRBJ1-5	13	199	17911	0.3869445
ATL26	M	54	Chronic	Indolent	78.14	TGTGCTA	CATGSNT	TRAV17	.	TRAJ37	12	TGTGCCT	CAWSVGV	TRBV30	.	TRBJ1-2	13	214	8827	0.5373731
ATL27	F	82	Chronic	Indolent	79.166	TGTGCAG	CAASMAA	TRAV13-1	.	TRAJ17	13	TGCAGTG	CSATQGQ	TRBV20-1	TRBD1	TRBJ2-3	12	59	25710	0.4418117
ATL29	M	76	Chronic	Indolent	NA	TGTGCTT	CAFKSRG	TRAV38-1	.	TRAJ57	14	TGTGCCA	CASCTPG	TRBV6-1	TRBD1	TRBJ2-7	16	506	9851	0.5935026
ATL32	M	74	Chronic	Indolent	60.658	TGTGTGG	CVVNMRY	TRAV12-1	.	TRAJ45	17	TGCAGTG	CSARARG	TRBV20-1	TRBD1	TRBJ2-1	20	906	13645	0.5808844
ATL34	F	50	Chronic	Indolent	87.361	TGTGCTC	CALGGGA	TRAV9-2	.	TRAJ54	13	TGTGCCA	CASSLEE	TRBV5-6	TRBD1	TRBJ1-2	15	347	7023	0.5739376
ATL36	M	70	Chronic	Indolent	78.658	TGTACTT	CTYGSSN	TRAV38-2	.	TRAJ37	14	TGTGCCT	CAWSPTG	TRBV30	TRBD1	TRBJ2-5	28	117	6541	0.298715
ATL37	M	81	Chronic	Indolent	75.541	TGTGTTG	CVVRFPF	TRAV1-2	.	TRAJ20	14	TGTGCCA	CASSEGL	TRBV6-1	TRBD2	TRBJ2-7	19	250	8593	0.4400302
ATL38	F	64	Chronic	Indolent	105.88	TGTGCTC	CALSGTS	TRAV9-2	.	TRAJ40	12	TGCAGTG	CSARGQN	TRBV20-1	TRBD1	TRBJ2-2	13	591	14290	0.5301976
ATL41	F	63	Chronic	Indolent	49.434	TGTGCTG	CAVRDSS	TRAV3	.	TRAJ3	13	TGTGCCA	CASSEDG	TRBV6-1	TRBD1	TRBJ1-6	13	58	1682	0.656032
ATL42	F	79	Chronic	Indolent	106.71	TGTGCTA	CATQMNA	TRAV17	.	TRAJ39	13	TGTGCCA	CASSWRT	TRBV28	TRBD1	TRBJ1-4	14	340	10660	0.5608602
ATL53	F	36	Chronic	Indolent	54.13	TGTGCAG	CAVQGGT	TRAV13-1	.	TRAJ44	13	TGTGCCA	CASSPRT	TRBV19	TRBD1	TRBJ1-5	16	562	5197	0.5900295
ATL66	F	69	Chronic	Indolent	70.857	TGTGCAG	CAARSTG	TRAV13-1	.	TRAJ9	13	TGTGCCA	CASSEVR	TRBV6-1	.	TRBJ1-1	13	157	1671	0.7724484
Kyo4	F	77	Chronic	Indolent	NA	TGTGCAG	CAGFNQG	TRAV25	.	TRAJ23	14	TGTGCCA	CASMRSG	TRBV2	TRBD1	TRBJ1-1	15	340	2242	0.8747295
Kyo8	F	64	Chronic	Indolent	NA	TGCATCC	CIPRDVG	TRAV26-2	.	TRAJ48	13	TGTGCCA	CASSLYR	TRBV14	TRBD1	TRBJ1-3	17	1178	4569	0.9293875
Kyo9	F	66	Chronic	Indolent	64.837	TGTGCTG	CAGMNT	TRAV8-3	.	TRAJ8	13	TGTGCCA	CASSYST	TRBV6-5	TRBD1	TRBJ1-1	12	171	863	0.8358538
Nag1	F	79	Chronic	Indolent	74.728	TGTGCAG	CAASMGN	TRAV13-1	.	TRAJ49	14	TGCAGTG	CSARLDG	TRBV20-1	.	TRBJ1-2	13	244	1931	0.6466416
Nag3	F	30	Chronic	Indolent	52.161	TGCATCG	CIVRANS	TRAV26-1	.	TRAJ41	15	TGTGCCA	CASSAET	TRBV6-1	TRBD1	TRBJ2-7	15	113	1704	0.6194504
Nag4	F	82	Chronic	Indolent	31.966	TGTGCAG	CAAKTGY	TRAV13-1	.	TRAJ3	15	TGTGCCA	CASSQSG	TRBV3-1	.	TRBJ2-6	13	146	1750	0.851742
Sas3	F	70	Chronic	Indolent	70.336	TGTGCAG	CAATGNQ	TRAV29D	.	TRAJ49	10	TGCAGTG	CSAKGGI	TRBV20-1	TRBD1	TRBJ1-5	12	89	724	0.6355499
Sas4	F	72	Chronic	Indolent	51.969	TGTGCTC	CALSFTG	TRAV9-2	.	TRAJ49	12	TGTGCCA	CASSLES	TRBV11-3	.	TRBJ2-1	15	125	1710	0.6653384
Sas5	F	80	Chronic	Indolent	NA	TGTGCTG	CAVSDYS	TRAV8-4	.	TRAJ45	16	TGCGCCA	CASSLETE	TRBV5-1	TRBD1	TRBJ1-1	14	247	2401	0.5972359
cd41	NA	NA	control	control	NA	NA	NA	NA	NA	NA	NA	NA	NA	NA	NA	NA	NA	2385	2793	0.9995181

cd42	NA	NA	control	control	NA	NA	NA	NA	NA	NA	NA	NA	NA	NA	NA	NA	NA	2422	3376	0.999476
cd43	NA	NA	control	control	NA	NA	NA	NA	NA	NA	NA	NA	NA	NA	NA	NA	NA	1973	2883	0.9993471
AC1	NA	NA	control	control	NA	NA	NA	NA	NA	NA	NA	NA	NA	NA	NA	NA	NA	1034	2018	0.9918862
AC3	NA	NA	control	control	NA	NA	NA	NA	NA	NA	NA	NA	NA	NA	NA	NA	NA	3309	4721	0.9992398
AC4	NA	NA	control	control	NA	NA	NA	NA	NA	NA	NA	NA	NA	NA	NA	NA	NA	466	1443	0.9965878
ATL02	F	64	Lymphoma	aggressive	144.46	TGTGCTG	CAVSERD	TRAV8-4	.	TRAJ30	12	TGTGCCA	CASSQAG	TRBV4-2	.	TRBJ2-3	13	418	16105	0.5269305
ATL39	F	59	Lymphoma	aggressive	46.262	TGTGCAG	CAAIFGNE	TRAV29DV	.	TRAJ48	11	TGCGCCA	CASNYEG	TRBV5-1	TRBD1	TRBJ1-1	14	247	4882	0.3758742
ATL43	M	67	Lymphoma	aggressive	79.299	TGTGCAG	CAASTGN	TRAV23DV	.	TRAJ49	14	TGTGCCA	CASSPGEA	TRBV9	TRBD1	TRBJ2-7	13	175	4200	0.1038022
NCC1	M	64	Lymphoma	aggressive	NA	TGTGCTG	CAVGRTG	TRAV2	.	TRAJ5	13	TGTGCCA	CASSPMG	TRBV7-2	TRBD1	TRBJ2-3	15	12	9818	0.431813
Sho1	M	53	Lymphoma	aggressive	82.601	TGTGCCG	CAVKFSG	TRAV12-2	.	TRAJ4	14	TGCAGTG	CSASTSGF	TRBV20-1	TRBD2	TRBJ2-3	14	11	4691	0.4842627
ATL33	F	61	Smoldering	Indolent	47.362	TGTGCTG	CAVNSGG	TRAV1-2	.	TRAJ13	13	TGTGCCA	CASSEGTT	TRBV6-1	TRBD2	TRBJ2-1	15	1478	5815	0.7262778
ATL40	F	36	Smoldering	Indolent	42.22	TGTGCAG	CAASNFN	TRAV13-1	.	TRAJ21	13	TGTGCCA	CASSIDRG	TRBV19	TRBD1	TRBJ1-2	13	1471	6858	0.903147
Kyo3	M	65	Smoldering	Indolent	19.272	TGTGCTG	CAVPFEGA	TRAV41	.	TRAJ54	12	TGTGCCT	CAWSRGS	TRBV30	.	TRBJ1-2	13	656	2407	0.910176
SNT22	M	62	Smoldering	Indolent	11.329	TGTGCTC	CALRHSG	TRAV38-1	.	TRAJ45	15	TGTGCCA	CASSVEGI	TRBV9	TRBD1	TRBJ2-1	15	263	420	0.9812585

reads*: "number of sequencing reads derived from repertoires", -AA: amino acid, -nu: nucleotide, -len: length

Supplementary Note-1: Supplementary methodology

Overview of the analysis pipeline: Each biological sample of blood or tissue will typically have thousands or millions of T/B-cell receptors (TCRs/BCRs) [1, 2]. This has prevented a global analysis of the full TCR/BCR repertoire using conventional DNA sequencing. The objective of a typical analysis pipeline within this context is to detect as completely as possible the arranged TCR/BCR gene sequences and to accurately determine their abundance within a sample[1].

Compared with conventional multiplex PCR, RNA-seq decreases the PCR bias that may result from different efficiencies of primers for different V and J genes [2].

Because clinical samples (blood or tissue) are often available in limited amounts, it is often not possible to split a sample for separate transcriptome and TCR/BCR profiling; extracting TCR/BCR transcripts that are present in bulk RNA-seq data is therefore highly advantageous.

Given that whole RNA-seq data contain only a small fraction of TCR/BCR reads (one per 10^5–10^7 reads) depending on the degree of immune cell infiltration/expansion, a reliable tool that enables sensitive and accurate extraction of clonotypes is necessary[1].

In the current study, we started from whole-transcriptome sequencing data prepared without any initial enrichment as a starting input in FASTQ format. In general, RNA-seq has a wide variety of applications, scientists plan experiments and optimize analysis workflows depending on their research goal [3]. In the current manuscript, we took advantage of an alternative workflow that enables simultaneous analysis and quantification of TCR and BCR profiles in a single high throughput sequencing assay. Currently MiXCR [1] is the most appropriate software for this kind of analysis [4]. MiXCR is a well-accepted tool for extracting BCR and TCR clonotypes from raw NGS

data. MiXCR allows millions of reads to be accurately and rapidly assigned to their respective variable V and J gene segments. MiXCR accepts either FASTA or FASTQ files as input. There is no limitation in the maximum size of the input file (the number of sequencing reads), and thus there is no need for adjusting the input size. MiXCR is distributed as a standalone program that works with the command line. A quality control procedure includes filtering and error correction steps that are built into the analysis pipeline. The ram requirement is 2 Gb. Average time of analysis for 10^8 reads is about 4 hours. There is no dependency on external software. MiXCR can analyze the TCR/BCR repertoire from different species including human, mouse and rat [1].

Data source origin: Genomic or transcriptomic data are acceptable for analysis by MiXCR [1]. In TCR/BCR profiling, using RNA (transcriptomic data) as the starting material is preferable to using DNA (genomic data), because RNA contains the final TCR/BCR transcripts. A uniform quality and quantity of starting material (RNA) also ensures a positive analysis outcome [2]. MiXCR, by default, assumes that the source of data is transcriptomic. MiXCR accepts sequencing input of different lengths (50-bp, 75-bp, 100-bp; paired end or single end), although 100-bp paired-end sequencing libraries can result in better yields (i.e., a higher number of clonotypes having fully matched CDR3s without mismatches or indels) [1].

Alignment: The FASTQ files need to be processed to result in meaningful biological information. This requires several stages of raw data processing. The first stage is to match the sequences to the known genomic reference (assign each TCR/BCR to its germline component gene sections). MiXCR performs this stage with its built-in alignment algorithm without requiring external alignment tools. MiXCR uses the KAligner algorithm, which is a particular version of the K-mer algorithm [1, 4].

MiXCR aligns the input data to the genomic sequences of V, D and J genes and filters out non-aligned reads before collecting the aligned reads.

Assembly: The assemble command builds clonotypes from alignments obtained with the built-in alignment algorithm. MiXCR assembles the reads containing fragments of CDR3. TCR sequences that contain defined V and J genes but do not fully cover the ends of the CDR3 region are extended using germline sequences.

Error correction: Error correction is a critical processing step that ensures the reliability of the final output. The common approach of trimming low-quality sequencing reads (based on the Phred quality score) is not suitable for the high-throughput analysis of TCR and BCR profiles. Because this approach typically removes only errors produced during base-calling, such an unsupervised quality filtration method can potentially be biased with respect to the removal of particular TCR/BCR rearrangements over others [5]. Therefore, for this kind of analysis, MiXCR uses a sophisticated error-editing approach based on clustering, through which MiXCR identifies similar sets of sequences and then absorbs the rarer member of each cluster into the more common ones. With this approach, MiXCR corrects the artificial diversity resulting from PCR and sequencing errors [1, 4, 6]. The default minimal value for the sequencing quality score is 20; nucleotides with a score of <20 are considered as "bad". If a sequencing read contains at least one bad nucleotide within the target gene region, it is left out of the initial assembly stage and is further processed by the mapper [1]. For further information, please refer to the MiXCR documentation in https://mixcr.readthedocs.io/en/master/.

Reporting the results: MiXCR reports the list of identified clonotypes, their abundance and VDJ gene composition. It can export all clonotypes as well as clonotypes for a

specific immunological chain. The tab-delimited output of MiXCR is parsable as input for the tcR and VDJ tools.

Accuracy and efficiency of analysis: RNA-seq TCR profiling is quantitative for clonotypes that make up >0.1% of the overall TCR repertoire. MiXCR can efficiently extract 90–100 TRB CDR3 reads per million unique reads. By analyzing 3×10^7 reads from 500 sorted T-cells, MiXCR can identify ~350–450 distinct clonotypes. This shows that MiXCR can accomplish almost complete repertoire extraction. In addition, software testing of MiXCR with data generated in silico has revealed a high extraction efficiency with no false positive clones observed [1].

The appropriate sequencing depth for each study depends on its goals. Deep sequencing is preferable for studies that aim to carry out an extensive analysis of the repertoire of cohorts and populations, whereas sequencing at a lower depth might be preferable for identification of already known or abundant clonotypes [2].

Terminology:

TCR profile: The sum of all TCRs present on the T cells of one individual.

Clonotype: A clonotype is a unique nucleotide sequence that arises during the gene rearrangement process for that receptor.

Spectratype: A histogram of clonotypes binned by CDR3 length and variable segment.

Supplementary References

1. Bolotin DA, Poslavsky S, Mitrophanov I, Shugay M, Mamedov IZ, Putintseva EV, Chudakov DM: **MiXCR: software for comprehensive adaptive immunity profiling**. *Nat Methods* 2015, **12**(5):380-381.
2. Rosati E, Dowds CM, Liaskou E, Henriksen EKK, Karlsen TH, Franke A: **Overview of methodologies for T-cell receptor repertoire analysis**. *BMC*

biotechnology 2017, **17**(1):61.

3. Conesa A, Madrigal P, Tarazona S, Gomez-Cabrero D, Cervera A, McPherson A, Szczesniak MW, Gaffney DJ, Elo LL, Zhang X *et al*: **A survey of best practices for RNA-seq data analysis**. *Genome biology* 2016, **17**:13.
4. Afzal S, Gil-Farina I, Gabriel R, Ahmad S, von Kalle C, Schmidt M, Fronza R: **Systematic comparative study of computational methods for T-cell receptor sequencing data analysis**. *Briefings in Bioinformatics* 2017, **20**(1):222-234.
5. Heather JM, Ismail M, Oakes T, Chain B: **High-throughput sequencing of the T-cell receptor repertoire: pitfalls and opportunities**. *Briefings in Bioinformatics* 2017, **19**(4):554-565.
6. Shugay M, Britanova OV, Merzlyak EM, Turchaninova MA, Mamedov IZ, Tuganbaev TR, Bolotin DA, Staroverov DB, Putintseva EV, Plevova K *et al*: **Towards error-free profiling of immune repertoires**. *Nat Methods* 2014, **11**(6):653-655.

Supplementary Note-2: Calculation of the Wilcoxon-Mann-Whitney test

Using the Wilcoxon-Mann-Whitney (WMW) test[1], we calculated *p*-values for differences in the number of clonotypes and in the Gini-Simpson index, a measure of diversity [2, 3], between different types of ATL (aggressive and indolent) as well as among different subtypes of ATL (smoldering, chronic, acute and lymphoma).

○Wilcoxon rank sum test with continuity correction

○Two-sided test

○W = *U*-value

Comparing the number of clonotypes

Supplementary Table-1

p-values for the number of clonotypes			
Type	Aggressive	Indolent	Control
Aggressive		0.003487	0.0002482
Indolent			0.001055
Control			

Supplementary Table-2

p-values for the number of clonotypes					
Subtype	Acute	Lymphoma	Chronic	Smoldering	Control
Acute		0.9254	0.01653	0.006218	0.0003775
Lymphoma			0.3555	0.03734	0.008113
Chronic				0.0138	0.0006828
Smoldering					0.1658
Control					

Raw clonotype data: **Aggressive and Indolent**

Group-1: Aggressive

583	81	148	373	145	38	280	40	23
123	14	685	149	186	137	134	15	42
245	219	41	52	418	247	175	12	11

Group-2: Indolent

1478	1471	656	263	149	189	199	214	59
506	906	347	117	250	591	58	340	562
157	340	1178	171	244	113	146	89	125
247								

W = 204, ***p*-value = 0.003487**

……………………………………………………………………………………………..

Raw clonotype data: Aggressive and Control

Group-1: Aggressive

583	81	148	373	145	38	280	40	23
123	14	685	149	186	137	134	15	42
245	219	41	52	418	247	175	12	11

Group-2: Control

2385	2422	1973	1034	3309	466

W = 2, ***p*-value = 0.0002482**

……………………………………………………………………………………………….

Raw clonotype data: **Indolent and Control**

Group-1: Indolent

1478	1471	656	263	149	189	199	214	59
506	906	347	117	250	591	58	340	562
157	340	1178	171	244	113	146	89	125
247								

Group-2: Control

2385	2422	1973	1034	3309	466

W = 11, ***p*-value = 0.001055**

Raw clonotype data: Chronic and Smoldering

Group-1: Chronic

149	189	199	214	59	506	906	347	117
250	591	58	340	562	157	340	1178	171
244	113	146	89	125	247			

Group-2: Smoldering

1478	1471	656	263

W = 10, ***p*-value = 0.0138**

……………………………………………………………………………………………..

Raw clonotype data: Acute and Chronic

Group-1: Acute

583	81	148	373	145	38	280	40	23
123	14	685	149	186	137	134	15	42
245	219	41	52					

Group-2: Chronic

149	189	199	214	59	506	906	347	117
250	591	58	340	562	157	340	1178	171
244	113	146	89	125	247			

W = 154.5, ***p*-value = 0.01653**

……………………………………………………………………………………………..

Raw clonotype data: Acute and Smoldering

Group-1: Acute

583	81	148	373	145	38	280	40	23
123	14	685	149	186	137	134	15	42
245	219	41	52					

Group-2: Smoldering

1478	1471	656	263

W = 5, ***p*-value = 0.006218**

………………………………………………………………………………………………

Raw clonotype data: Acute and Control

Group-1: Acute

583	81	148	373	145	38	280	40	23
123	14	685	149	186	137	134	15	42
245	219	41	52					

Group 2: Control

2385	2422	1973	1034	3309	466

W = 2, ***p*-value = 0.0003775**

…………………………………………………………………………………………………

Raw clonotype data: Smoldering and Control

Group-1: Smoldering

1478	1471	656	263

Group-2: Control

2385	2422	1973	1034	3309	466

W = 5, ***p*-value = 0.1658**

………………………………………………………………………………………………..

Raw clonotype data: **Chronic and Control**

Group-1: Chronic

149	189	199	214	59	506	906	347	117
250	591	58	340	562	157	340	1178	171
244	113	146	89	125	247			

Group-2: Control

2385	2422	1973	1034	3309	466

W = 6, ***p*-value = 0.0006828**

……………………………………………………………………………………………….

Raw clonotype data: Lymphoma and Control

Group-1: Lymphoma

418	247	175	12	11

Group-2: Control

2385	2422	1973	1034	3309	466

W = 0, **p-value = 0.008113**

Raw clonotype data: Lymphoma and Acute

Group-1: Lymphoma

418	247	175	12	11				

Group-2: Acute

583	81	148	373	145	38	280	40	23
123	14	685	149	186	137	134	15	42
245	219	41	52					

W = 53, **p-value = 0.9254**

……………………………………………………………………………………………………

Raw clonotype data: Lymphoma and Chronic

Group-1: Lymphoma

418	247	175	12	11				

Group-2: Chronic

149	189	199	214	59	506	906	347	117
250	591	58	340	562	157	340	1178	171
244	113	146	89	125	247			

W = 43.5, ***p*-value = 0.3555**

…………………………………………………………………………………………………

Raw clonotype data: Lymphoma and Smoldering

Group-1: Lymphoma

418	247	175	12	11

Group-2: Smoldering

1478	1471	656	263

W = 1, ***p*-value = 0.03734**

Comparing the Gini-Simpson index

Supplementary Table-3

p-values for the Gini-Simpson index			
Type	Aggressive	Indolent	Control
Aggressive		0.0002855	0.0001717
Indolent			0.0001618
Control			

Supplementary Table-4

p-values for the Gini-Simpson index					
Subtype	Acute	Lymphoma	Chronic	Smoldering	Control
Acute		0.2482	0.01257	0.002525	0.0002451
Lymphoma			0.005108	0.01996	0.008113
Chronic				0.007838	0.0002096
Smoldering					0.01421
Control					

Raw index data: Aggressive and Indolent

Group-1: Aggressive

0.533358422	0.466700904	0.558351383	0.812571093
0.333803548	0.478173285	0.590558126	0.446603488
0.301096313	0.48735022	0.322672971	0.607884196
0.339910104	0.690237484	0.30308845	0.582673895
0.502351965	0.726061595	0.658103643	0.210691729
0.403493607	0.491283661	0.526930516	0.375874153
0.103802154	0.431813041	0.484262716	

Group-2: Indolent

0.634609101	0.605038995	0.386944504	0.537373122
0.441811686	0.593502635	0.580884446	0.573937561
0.298714954	0.440030234	0.530197611	0.656032044
0.560860171	0.590029497	0.772448367	0.874729537
0.929387532	0.835853802	0.646641606	0.619450368
0.851742041	0.635549892	0.665338395	0.59723588
0.726277772	0.903147004	0.910175953	0.981258503

W = 162, **p-value = 0.0002855**

Raw index data: Aggressive and Control

Group-1: Aggressive

0.533358422	0.466700904	0.558351383	0.812571093
0.333803548	0.478173285	0.590558126	0.446603488
0.301096313	0.48735022	0.322672971	0.607884196
0.339910104	0.690237484	0.30308845	0.582673895
0.502351965	0.726061595	0.658103643	0.210691729
0.403493607	0.491283661	0.526930516	0.375874153
0.103802154	0.431813041	0.484262716	

Group-2: Control

0.999518129	0.99947602	0.999347064	0.991886206
0.99923981	0.996587824		

W = 0, **p-value = 0.0001717**

…………………………………………………………………………………………………….

Raw index data: Indolent and Control

Group-1: Indolent

0.634609101	0.605038995	0.386944504	0.537373122
0.441811686	0.593502635	0.580884446	0.573937561
0.298714954	0.440030234	0.530197611	0.656032044
0.560860171	0.590029497	0.772448367	0.874729537
0.929387532	0.835853802	0.646641606	0.619450368
0.851742041	0.635549892	0.665338395	0.59723588
0.726277772	0.903147004	0.910175953	0.981258503

Group-2: Control

0.999518129	0.99947602	0.999347064	0.991886206
0.99923981	0.996587824		

W = 0, ***p*-value = 0.0001618**

………………………………………………………………………………………………

Raw index data: Chronic and Smoldering

Group-1: Chronic

0.634609101	0.605038995	0.386944504	0.537373122
0.441811686	0.593502635	0.580884446	0.573937561
0.298714954	0.440030234	0.530197611	0.656032044

0.560860171	0.590029497	0.772448367	0.874729537
0.929387532	0.835853802	0.646641606	0.619450368
0.851742041	0.635549892	0.665338395	0.59723588

Group-2: Smoldering

0.726277772	0.903147004	0.910175953	0.981258503

W = 7, ***p*-value = 0.007838**

……………………………………………………………………………………………….

Raw index data: Acute and Chronic

Group-1: Acute

0.533358422	0.466700904	0.558351383	0.812571093
0.333803548	0.478173285	0.590558126	0.446603488
0.301096313	0.48735022	0.322672971	0.607884196
0.339910104	0.690237484	0.30308845	0.582673895
0.502351965	0.726061595	0.658103643	0.210691729
0.403493607	0.491283661		

Group-2: Chronic

0.634609101	0.605038995	0.386944504	0.537373122
0.441811686	0.593502635	0.580884446	0.573937561
0.298714954	0.440030234	0.530197611	0.656032044
0.560860171	0.590029497	0.772448367	0.874729537
0.929387532	0.835853802	0.646641606	0.619450368
0.851742041	0.635549892	0.665338395	0.59723588

W = 150, ***p*-value = 0.01257**

……………………………………………………………………………………………….

Raw index data: Acute and Smoldering

Group-1: Acute

0.533358422	0.466700904	0.558351383	0.812571093
0.333803548	0.478173285	0.590558126	0.446603488
0.301096313	0.48735022	0.322672971	0.607884196
0.339910104	0.690237484	0.30308845	0.582673895
0.502351965	0.726061595	0.658103643	0.210691729
0.403493607	0.491283661		

Group-2: Smoldering

0.726277772	0.903147004	0.910175953	0.981258503

W = 1, ***p*-value = 0.002525**

……………………………………………………………………………………………….

Raw index data: Acute and Control

Group-1: Acute

0.533358422	0.466700904	0.558351383	0.812571093
0.333803548	0.478173285	0.590558126	0.446603488
0.301096313	0.48735022	0.322672971	0.607884196
0.339910104	0.690237484	0.30308845	0.582673895
0.502351965	0.726061595	0.658103643	0.210691729
0.403493607	0.491283661		

Group-2: Control

0.999518129	0.99947602	0.999347064	0.991886206
0.99923981	0.996587824		

W = 0, ***p*-value = 0.0002451**

……………………………………………………………………………………………….

Raw index data: Smoldering and Control

Group-1: Smoldering

0.726277772	0.903147004	0.910175953	0.981258503

Group-2: Control

0.999518129	0.99947602	0.999347064	0.991886206
0.99923981	0.996587824		

W = 0, ***p*-value = 0.01421**

……………………………………………………………………………………………….

Raw index data: Chronic and Control

Group-1: Chronic

0.634609101	0.605038995	0.386944504	0.537373122
0.441811686	0.593502635	0.580884446	0.573937561
0.298714954	0.440030234	0.530197611	0.656032044
0.560860171	0.590029497	0.772448367	0.874729537
0.929387532	0.835853802	0.646641606	0.619450368
0.851742041	0.635549892	0.665338395	0.59723588

Group-2: Control

0.999518129	0.99947602	0.999347064	0.991886206
0.99923981	0.996587824		

W = 0, ***p*-value = 0.0002096**

……………………………………………………………………………………………….

Raw index data: Lymphoma and Control

Group-1: Lymphoma

0.526930516	0.375874153	0.103802154	0.431813041
0.484262716			

Group-2: Control

0.999518129	0.99947602	0.999347064	0.991886206
0.99923981	0.996587824		

W = 0, ***p*-value = 0.008113**

…………………………………………………………………………………………………

Raw index data: Lymphoma and Acute

Group-1: Lymphoma

0.526930516	0.375874153	0.103802154	0.431813041
0.484262716			

Group-2: Acute

0.533358422	0.466700904	0.558351383	0.812571093
0.333803548	0.478173285	0.590558126	0.446603488
0.301096313	0.48735022	0.322672971	0.607884196
0.339910104	0.690237484	0.30308845	0.582673895
0.502351965	0.726061595	0.658103643	0.210691729
0.403493607	0.491283661		

W = 36, ***p*-value = 0.2482**

Raw index data: Lymphoma and Chronic

Group-1: Lymphoma

0.526930516	0.375874153	0.103802154	0.431813041
0.484262716			

Group-2: Chronic

0.634609101	0.605038995	0.386944504	0.537373122
0.441811686	0.593502635	0.580884446	0.573937561
0.298714954	0.440030234	0.530197611	0.656032044
0.560860171	0.590029497	0.772448367	0.874729537
0.929387532	0.835853802	0.646641606	0.619450368
0.851742041	0.635549892	0.665338395	0.59723588

W = 11, ***p*-value = 0.005108**

……………………………………………………………………………………………

Raw index data: Lymphoma and Smoldering

Group-1: Lymphoma

0.526930516	0.375874153	0.103802154	0.431813041
0.484262716			

Group-2: Smoldering

0.726277772	0.903147004	0.910175953	0.981258503

W = 0, ***p*-value = 0.01996**

Supplementary References

1. Fay MP, Proschan MA: Wilcoxon-Mann-Whitney or t-test? On assumptions for hypothesis tests and multiple interpretations of decision rules. *Statistics surveys* 2010, **4**:1-39.
2. Kaplinsky J, Arnaout R: Robust estimates of overall immune-repertoire diversity from high-throughput measurements on samples. *Nature communications* 2016, **7**:11881-11881.
3. Nazarov VI, Pogorelyy MV, Komech EA, Zvyagin IV, Bolotin DA, Shugay M, Chudakov DM, Lebedev YB, Mamedov IZ: tcR: an R package for T cell receptor repertoire advanced data analysis. *BMC Bioinformatics* 2015, **16**:175.
